# Risk factors for position-related injuries in adult surgical patients: a systematic review and meta-analysis

**DOI:** 10.1016/j.ijnsa.2026.100676

**Published:** 2026-09-08

**Authors:** Veronica Ramirez Johansson, Oili Dahl, Ann-Charlotte Falk, Ulrica Nilsson, Marina Dehara, Ann-Christin von Vogelsang

**Affiliations:** a Department of Clinical Neuroscience, Karolinska Institutet, Stockholm, Sweden; b Perioperative Medicine and Intensive Care Function, Karolinska University Hospital, Stockholm, Sweden; c Department of Neurobiology, Care Sciences and Society, Division of Nursing, Karolinska Institutet, Stockholm, Sweden; d Swedish Society of Nursing, Stockholm, Sweden; e Department of Medicine, Clinical Epidemiology Division, Karolinska Institutet, Stockholm, Sweden; f Department of Neurosurgery, Karolinska University Hospital, Stockholm, Sweden

**Keywords:** Patient positioning, Peripheral nerve injuries, Pressure injuries, Risk factors, Surgery

## Abstract

**Background:**

Correct patient positioning is a vital responsibility of the operating team to ensure patient safety. By identifying risk factors associated with position-related injuries in surgical patients, perioperative nurses can develop effective strategies to prevent these iatrogenic injuries

**Objective:**

To identify and synthesise intrinsic and extrinsic risk factors for position-related pressure injuries and peripheral nerve injuries.

**Information sources:**

Searches were conducted in Medline, Web of Science, CINAHL, Embase, and the Cochrane Library (January 1990 – September 2025).

**Methods:**

Observational studies examining intrinsic and extrinsic risk factors for new pressure injuries or peripheral nerve injuries in adult surgical patients were included. The risk of bias was assessed using the Research Triangle Institute item bank framework. Meta-analyses were performed using random-effects models, with sensitivity analyses regarding study design and risk of bias.

**Results:**

In total, 16 382 records were screened, and 161 full-text articles were assessed for eligibility. Of these, 41 studies were included (33 on pressure injuries comprising 26 074 patients and 8 on peripheral nerve injuries comprising 1 338 592 patients), assessing 101 potential risk factors. Four factors were found to be statistically significant in association with pressure injuries: diabetes (OR 1.89; 95% CI 1.25–2.86), high body mass index (MD 1.80; 95% CI 0.38 to 3.22), low preoperative serum albumin (MD -3.04; 95% CI - 4.99 to -1.09), and intraoperative use of circulatory agents (OR 2.11; 95% CI 1.13 to 3.92). Biological sex (OR 1.10; 95% CI 0.94–1.30), age (MD 0.69; 95% CI -1.65 to 3.03), hypertension (OR 1.37; 95% CI 0.93–2.01), low haemoglobin (MD -7.64; 95% CI -16.90 to 1.62), and surgery duration (MD 42.54; 95% CI -9.03 to 94.11) were not associated with injury risk. For peripheral nerve injuries, the available evidence on risk factors was too limited and heterogenous to permit meta-analysis.

**Conclusions:**

Surgical patients are at risk of position-related injuries, particularly in the presence of factors such as diabetes, high body mass index, low serum albumin, and use of circulatory agents. Preoperative optimisation and thorough postoperative assessment are essential to reduce these iatrogenic injuries.

**Registration:**

www.crd.york.ac.uk/prospero/ PROSPERO CRD42023401329, registered 21/02/2023.

**What is already known**

• Safe patient positioning during surgery is essential to prevent position-related injuries
• Various intrinsic and extrinsic risk factors elevate the risk of patient injury

**What this paper adds**

• This review highlights key factors associated to surgical position-related injuries
• Findings show inconsistent injury reporting, affecting clinical evaluation
• Insights may strengthen nursing practice in perioperative risk prevention

## 1 Background

Patient safety is defined as the reduction of the risk of unnecessary harm associated with healthcare to an acceptable minimum. However, around one in every ten patients is harmed in healthcare, most commonly as a result of adverse events during hospitalisation (WHO, 2023). The operating room is a critical setting where the surgical patients are exposed to unexpected events that also contribute to unplanned extrinsic and perioperative risk factors like bleeding or prolonged surgery time (Bulfone et al., 2018).

In this review, the term position-related injuries is used to describe injuries resulting from placing and maintaining patients in a surgical position on the operating table. These are well-known complications that may occur despite adequate preventive measures (Grant et al., 2019; Haisley et al., 2020), and all members of the surgical team share the responsibility for the safe patient positioning (Sørensen et al., 2016). Within perioperative nursing, patient advocacy is a key element, with the aim to ensure the best and safest outcome for the patient. This includes safeguarding the patient from harm by proactively identifying and minimising extrinsic risks that may affect the patient during surgery (Sundqvist et al., 2018).

Previous research has described the presence of several types of position-related injuries including pressure injuries, peripheral nerve injuries (Hewson et al., 2018), lower extremity compartment syndrome (Mizuno et al., 2023), deep venous thrombosis (Gordon and Lombard, 2017) and posterior ischemic optic neuropathy (Lee et al., 2010), even though some of these complications are rare. These injuries result from immobility and type of surgical position, but are also influenced by, e.g., anaesthesia, which reduces or eliminates the patient’s ability to perceive pain and pressure (Sørensen et al., 2016).

Pressure injuries are a common complication among surgical patients, with a mean incidence of 17.2% reported in a recent systematic review (Kurian et al., 2025), with variations among surgical specialties (Fang et al., 2025; Ramezanpour et al., 2018). A pressure injury is defined as localised damage to the skin and/or underlying soft tissue, usually over a bony prominence and/or related to a medical or other device, resulting from intense and/or prolonged pressure, or pressure in combination with shear. Pressure injuries are classified as Stage 1–4, Unstageable pressure injury, and Deep Tissue Pressure Injury (Edsberg et al., 2016).

Comparably, the incidence of peripheral nerve injuries related to surgical positioning is lower, varies between surgical specialties, and is affected by the surgical techniques where different patient positions are needed. The incidence is, for instance, estimated to 0.2% in open colorectal surgery (Navarro-Vicente et al., 2012) and up to 10.8% in robotic urological surgery (Mills et al., 2013). Robotic or laparoscopic surgery in the pelvic area often requires a more advanced position, where the body is tilted with the head down and with abducted and flexed legs in stirrups (Bjøro et al., 2020; Cornelius et al., 2021). A peripheral nerve injury is a sensory and/or motor deficit, and the aetiology of this type of injury is multifactorial. The most reported underlying mechanisms include stretch, compression, hypoperfusion, and metabolic effects (Hewson et al., 2018). The severity of a nerve injury varies, and recovery can take from a few days to over a year. Symptoms such as numbness, tingling, and impaired motor function in the extremities may impede the patient’s recovery after surgery (Bouyer-Ferullo, 2013; Hewson et al., 2018). In the most severe cases, the injury does not resolve and results in a permanent deficit (Bouyer-Ferullo, 2013). According to the literature, the true incidence of peripheral nerve injuries is difficult to determine due to underreporting (Mills et al., 2013) and because symptoms can appear days or even weeks after surgery (Hewson et al., 2018).

The literature identifies a large number of potential risk factors associated with the development of pressure injuries (Fang et al., 2025) and peripheral nerve injuries (Al-Temimi et al., 2017) including both patient-related (intrinsic) and operative/environmental (extrinsic) factors (Özdemir et al., 2023). In studies on pressure injuries, more than 100 potential risk factors have been reported, such as comorbidities including diabetes and cardiovascular disease (Haisley et al., 2020). Regarding peripheral nerve injuries, increasing age, smoking, and diabetes have been identified as risk factors associated with their development (Welch et al., 2009).

However, different studies claim various risk factors to be statistically significant for position-related injuries in surgical patients (Haisley et al., 2020; Welch et al., 2009) and presented risk factors without multivariable analyses limit both interpretations and conclusions (Coleman et al., 2013). A previous systematic review on intrinsic risk factors for pressure injuries reported methodological limitations in observational studies, including reporting bias and insufficient examination of potential confounding variables. Therefore, findings from observational studies require careful consideration (Coleman et al., 2013), and meta-analyses that combine results from multiple studies may help identify the most statistically significant risk factors.

For perioperative nurses and other practising clinicians caring for patients pre-, intra-, and postoperatively, an improved understanding of risk factors to identify patients at high risk for position-related injuries in connection with surgery is essential (Al-Temimi et al., 2017; Celik et al., 2019). Preventing pressure injuries is a crucial indicator of care quality, and nursing interventions play an important role in both preventing and reducing their occurrence (Yılmaz and Başlı, 2021). Evidence-based information on which risk factors that need to be taken into consideration enables optimisation beforehand, together with the planning of specific actions in connection with surgical positioning (Coleman et al., 2013).

Although pressure injuries and peripheral nerve injuries differ in their clinical manifestations and pathophysiology, both are recognised as position-related injuries that may arise from prolonged pressure, tissue deformation, stretching, and impaired perfusion during surgery. Furthermore, perioperative positioning practices and preventive strategies are often implemented concurrently to reduce the risk of both injury types (Burlingame, 2017). Examining both outcomes within the same systematic review therefore provides a broader understanding of position-related injuries while allowing each outcome to be analysed separately. No systematic review has synthesised the available evidence on intrinsic and extrinsic risk factors for these injuries. To address this knowledge gap, the present systematic review and meta-analysis aimed to identify and synthesise intrinsic and extrinsic risk factors for position-related pressure injuries and peripheral nerve injuries.

## 2 Methods

### 2.1 Design

This systematic review is reported in accord with the PRISMA reporting guideline (Page et al., 2021). The systematic review has been registered in the International Prospective Register of Systematic Reviews (PROSPERO), https://www.crd.york.ac.uk/prospero/, ID: CRD42023401329.

### 2.2 Search methods

Comprehensive literature searches of scientific publications were performed and were last updated on the 11th of September 2025. The search strategy was developed in collaboration with the librarians at the Karolinska Institutet University Library, including publications from January 1990 to September 2025. Five databases; Medline (Ovid), CINAHL (EBSCOhost), Web of Science (Clarivate), Embase (Ovid) and Cochrane Central Register of Controlled Trials (Ovid) - were searched to identify relevant publications. Key search terms were “surgery”, “risk factor”, “pressure injury”, “pressure ulcer”, and “peripheral nerve injury”. The search strategy was designed to maximize the retrieval of studies on position-related injuries by combining terms for both pressure and peripheral nerve injuries in a single, broad search, which also identified older publications. Handsearching was performed by scanning all reference lists of the articles included in the study for the identification of additional publications of relevance not captured in the database search. The complete search strategy is shown in PROSPERO, https://www.crd.york.ac.uk/prospero/, ID: CRD42023401329.

### 2.3 Inclusion and exclusion criteria

Studies were selected based on the following inclusion criteria:

(1) Adult surgical populations in any inpatient setting
(2) Investigation of risk factors for intraoperative position-related pressure injuries or peripheral nerve injuries
(3) Reported occurrence of pressure injuries or peripheral nerve injuries related to the surgical occasion
(4) Observational study design

#### 2.3.1 Further eligibility specifications

Comparisons between pressure injury- and/or peripheral nerve injury versus no injury were required, and injuries had to be clinically assessed by medical staff or be documented in patients’ medical records. All categories of pressure injuries and all types of peripheral nerve injuries, as well as all pre- and intraoperative risk factors studied in any surgical setting under general and/or regional anaesthesia, were eligible. Pressure injury assessment was required to be based on a recognised international pressure injury classification system. No specified follow-up time was required for pressure injury assessment. For peripheral nerve injuries, a new sensory or motor deficit within 48 h (Welch et al., 2009) and a subsequent assessment at ≥14 days (Hewson et al., 2018) were required. Eligible studies were limited to publications in English or Scandinavian languages.

### 2.4 Exclusion criteria

The following exclusion criteria were applied: studies including patients under 18 years of age in the group where injuries had occurred; studies including patients with pre-existing pressure- or peripheral nerve injury; patients with neurological diseases at admission to the hospital; studies focusing on compartment syndrome, ischemic optic neuropathy, or assessing somatosensory evoked potentials without associated clinical symptoms.

Further exclusions were applied to studies evaluating pressure- or peripheral nerve injuries caused by surgical technique or dental procedures; studies including ambulatory surgery or reporting multiple surgeries during the same inpatient admission; studies involving patients treated in intensive care units where the cause of pressure injury development was unclear; studies including medical-device pressure injuries, and studies comparing different positioning materials.

### 2.5 Study selection and data extraction

The selection process was conducted by two independent persons in the research group, with the first author in every constellation of assessors. The Rayyan web application was used to support the review process, enabling blinded individual screening of articles (Ouzzani et al., 2016). Potentially eligible articles according to the abstracts were retrieved, and the articles were read in full text for assessment of eligibility based on the inclusion and exclusion criteria. Corresponding authors of three studies were contacted for further information (Celik et al., 2019; Yoshimura et al., 2015; Luo et al., 2019). Disagreements regarding inclusion were resolved by consensus among the group of five reviewers.

The extraction of data items was carried out by the first author and was thereafter assessed by three other authors in the research group in pairs with the first author. Bibliographic data, i.e. authors, year of publication, and country was extracted and put in a separate data extraction form. Further, the following data was collected: aim, study design, sample size, incidence, studied risk factors and used data analyses (univariable or multivariable analysis). Although both outcomes were included within the broader concept of position-related injuries, pressure injuries and peripheral nerve injuries were treated as separate outcomes during data extraction, evidence synthesis, and reporting. Data of specific interest for the study outcomes, such as surgical setting, body location of the injury, classification of pressure injuries and type of surgical position were also extracted. Identified risk factors and their association with injury was the primary outcome, and adjusted estimates were extracted from each study. When an adjusted estimate was not reported or no specific model was provided for the association of interest, calculations of the crude effect estimate were performed along with their corresponding 95% confidence interval (CI).

### 2.6 Risk of bias assessment

The included studies were assessed for risk of bias and confounding using an adapted version of the further development of the Research Triangle Institute (RTI) Item Bank (Viswanathan et al., 2013). The RTI item bank contains 16 items including an overall assessment and is intended to be adapted to the review question and study designs. Accordingly, three items (Q5, Q10 and Q16) were considered not applicable and excluded. Following these modifications, 13 items including an overall assessment remained. Overall risk of bias judgments were based on a qualitative assessment of all applicable items rather than a numerical score, considering both the number of items rated as having a high risk of bias and the extent to which these items could affect the overall validity of each study. Risk of bias assessment was performed independently by four of the researchers. Disagreements were first resolved in pairs, and any remaining disagreements were resolved by consensus within the research group.

### 2.7 Data synthesis and statistical analysis

Primarily, a descriptive approach was used with tabular summaries to organise the reported findings. Pressure injuries and peripheral nerve injuries were synthesised and reported separately. Meta-analysis was performed when six or more studies, reported on the same risk factor with comparable outcome measures for a valid interpretation of the results (Davey et al., 2011). The results of the meta-analyses are presented as Odds Ratios (ORs) with 95% CI for dichotomous variables and mean differences (MDs) for continuous variables. Adjusted effect estimates were used when reported in the original studies; otherwise, unadjusted estimates were extracted. Where necessary, measurement units for continuous laboratory variables were standardised before quantitative synthesis to ensure consistency across studies. Albumin and haemoglobin concentrations reported in different units were converted to a common unit before pooling. Random-effects meta-analysis using restricted maximum likelihood (REML) was performed for all variables to account for expected clinical and methodological heterogeneity across studies. For age, the Paule–Mandel estimator was used due to convergence difficulties with standard REML. Heterogeneity was tested using the Q-statistic (considered statistically significant if p < 0.10), and by calculating the I² (the percent of total variability due to between-study heterogeneity) and p-value. I² > 75% indicates that a high percentage of the total variance in effect estimates is due to heterogeneity rather than chance. Heterogeneity was also estimated by calculating the between-study variance, τ2.

Sensitivity analyses were performed by excluding studies with high risk of bias to evaluate whether studies with lower quality affected the results. Additional sensitivity analyses were performed by excluding all retrospective and case-control studies to investigate potential sources of heterogeneity due to study design. For diabetes, hypertension, and intraoperative use of circulatory agents, the primary meta-analyses combined crude and adjusted estimates. Additional sensitivity analyses were therefore performed using crude estimates only to assess the robustness of the pooled effect estimates as only a limited number of studies reported adjusted estimates, and these were based on substantially different adjustment models, precluding a separate meta-analysis using adjusted estimates. Moreover, the robustness of the results was evaluated by investigating whether the pooled estimates were influenced by the removal of each individual study (influence analysis). To examine the presence of publication bias, visual evaluation of the symmetry of funnel plots for risk factors was made (Higgins et al., 2019). In addition, Egger’s test was used to examine publication bias and small-study effects (considered statistically significant if p < 0.05). As a rule of thumb, tests for funnel plot asymmetry should only be used when at least 10 studies are included in the meta-analysis due to the low power when fewer studies are included (Sterne et al., 2011). No formal certainty grading approach was applied; overall confidence in the body of evidence was judged based on the risk-of-bias assessments and the consistency, heterogeneity, and precision of the meta-analyses.

Data management and all statistical analyses were performed using STATA (StataCorp. 2023. Stata Statistical Software: Release 18. College Station, TX: StataCorp LLC).

## 3 Results

### 3.1 Literature search

**Fig. 1:**
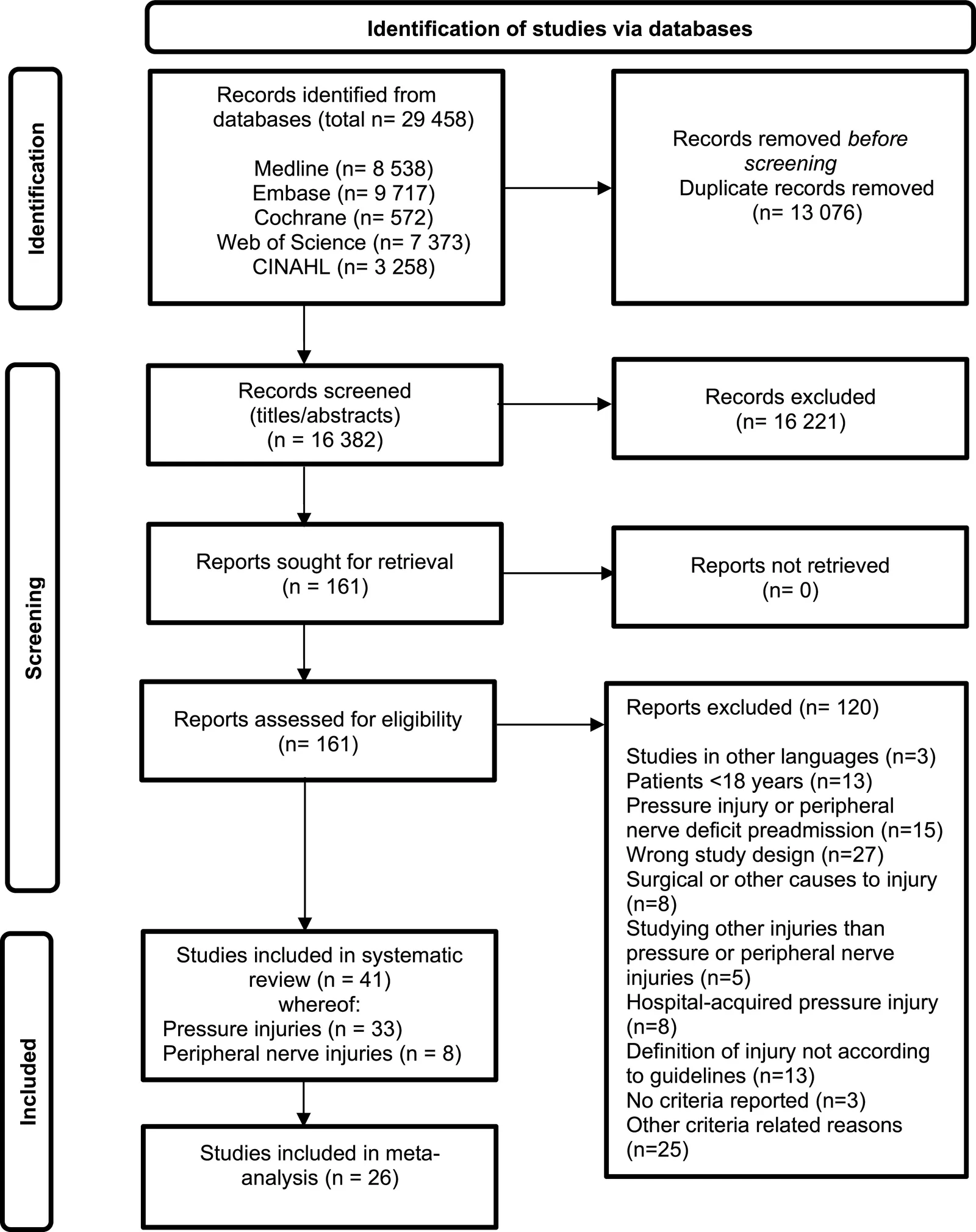
Flow diagram of the study identification, screening, eligibility assessment, and inclusion in the systematic review and meta-analysis.

The database searches yielded 16 382 citations after deduplication, and 161 publications were reviewed in full text. A total of 41 publications were included in the systematic review, of which 33 addressed pressure injuries and 8 addressed peripheral nerve injuries. No further study was detected when handsearching the reference lists. Among the included studies on pressure injuries, 26 contributed to the meta-analyses (Fig. 1). No meta-analyses were performed for risk factors for peripheral nerve injuries due to insufficient and non-comparable numerical data across studies and inadequate number per risk factor to justify pooling.

### 3.2 Study characteristics and incidence of reported injuries

Study characteristics for pressure injuries and peripheral nerve injuries are summarised in Table 1. The geographical distribution differed between the two groups. Among the 33 studies on pressure injuries, 16 were conducted in Asia of which 9 originated from China. An additional 7 studies were performed in Turkey, and the remaining studies were conducted in various other countries without any clear geographical clustering. Among the 8 studies on peripheral nerve injuries, 6 originated from the United States with the remainder conducted in other countries. Regarding study design, the pressure injury literature was dominated by cross-sectional studies with follow-up during the inpatient period (n = 20). In addition, 2 studies used a case-control design, and 13 were retrospective register-based studies.

**Table 1:** Study characteristics of included studies on position-related injuries.

Reference and country	Study design	Surgical specialty	Surgical position	Sample size	Patients with injury, n (%)	Injury-specific outcome	Follow-up
*Pressure injuries*							
Bulfone et al., 2012 Italy	Cross-sectional	General, cardiac, vascular, plastic- and neurosurgery	Supine, lateral, prone, kneeling, and lithotomy	102	13 (12.7)	-	Directly after surgery, postop day 3 and 6
Buso et al., 2021 Brazil	Cross-sectional	Not specified	Supine, lateral, Trendelenburg, prone, and lithotomy	239	90 (37.7)	-	Directly after surgery, 24, 48 and up to 72 h after surgery
Celik et al., 2019 Turkey	Cross-sectional	Neuro, abdominal, thoracic, and cardiovascular surgery	Supine and prone	151	61 (40.4)	-	Daily, for up to 72 h after surgery
Chen et al., 2017 China	Retrospective cohort	Hepato-biliary surgery	Supine	803	159 (19.8)	-	Directly after surgery, postoperative day 1 and 2
Chen et al., 2019 China	Retrospective cohort	Liver surgery	Not described	128	11 (8.6)	-	Not described
Chen et al., 2020 China	Retrospective cohort	Cardiovascular surgery	Supine	214	43 (20.1)	-	Not described
Chen et al., 2024 Taiwan	Retrospective cohort	Not specified	Supine, lithotomy, sitting, semi-sitting, lateral, jackknife, and prone	11 231	250 (2.2)	-	Not described
Choi et al., 2024 South Korea	Cross-sectional	Spine surgery	Prone	287	71 (24.7)	-	Directly after surgery, postoperative day 1 and 2
Connor et al., 2010 USA	Cross-sectional	Urology surgery	Not described	498	25 (5.0)	-	At postop. unit and daily, for up to 72 h after surgery
Dong et al., 2021 China	Case-control	Aortic surgery	Supine and lateral	400	167 (41.8)	-	Not described
Forni et al., 2021 Italy	Retrospective cohort	Knee replacement	Not described	565	13 (2.3)	-	Unclear. Review of the patients’ medical records until discharge
İlkhan & Sucu Dag, 2023 Turkey	Cross-sectional	General surgery, orthopaedic and cardiovascular surgery	Not described	342	56 (16.4)	-	Unclear. Up to 72 h after surgery
Kandemir et al., 2022 Turkey	Cross-sectional	General surgery	Not described	101	13 (12.9)	-	At postoperative unit and daily up to 72 h after surgery
Karadede et al., 2025 Turkey	Cross-sectional	Cardiovascular, general, urological, and orthopaedic surgery	Not described	158	22 (13.9)	-	Daily for three days postoperatively
Karahan et al., 2022 Turkey	Cross-sectional	General, neuro- and gynaecological surgery	Supine, lateral, prone, and lithotomy	250	32 (12.8)	-	Directly after surgery and within 24 h after surgery
Kemp et al., 1990 USA	Cross-sectional	General, cardiovascular, orthopaedic, transplant, head and neck, thoracic, neuro, gynaecologic and urological surgery	Not described	125	15 (12.0)	-	Postoperative day 1, 4, 7 and 10
Lei et al., 2022 China	Case-control	Any surgical specialty	Not described	126	42	-	Unclear. Within 72 h after surgery
Lewicki et al., 1997 USA	Cross-sectional	Cardiac surgery	Not described	337	16 (4.7)	-	Directly after surgery, postop. day 1, 3 and 5
Li et al., 2025 China	Retrospective cohort	Prone position surgery	Prone	970	173 (17.8)	-	Not described
Luo et al., 2019 China	Retrospective cohort	Spinal surgery	Prone	3 840	184 (4.7)	-	Not described
Ninlaphut et al., 2025 Thailand	Retrospective cohort	Spinal surgery	Prone	590	159 (26.9)	-	Not described
Peng et al., 2024 China	Retrospective cohort	Neurosurgery	Supine, prone, and lateral	1 728	31 (1.8)	-	Directly after surgery
Ramezanpour et al., 2018 Iran	Cross-sectional	General surgery	Not described	191	34 (17.8)	-	Every 24 h, up to 72 h after surgery
Scarlatti et al., 2011 Brazil	Cross-sectional	Not specified	Supine, prone, lateral, Fowler and lithotomy	199	41 (20.6)	-	Postoperative day 1
Shaw et al., 2014 Taiwan	Cross-sectional	Cardiovascular, general, chest, orthopaedic, neuro, plastic, and urological surgery	Supine, prone, lithotomy, lateral, and others	297	15 (5.1)	-	Directly after surgery and 30 min postoperatively
Shi et al., 2023 China	Cross-sectional	General, orthopaedic, urology, neurosurgery and other unspecific	Supine, prone, lateral, cystectomy	413	43 (10.4)	-	Not described
Suh et al., 2021 South Korea	Retrospective cohort	Spine surgery	Not specified	663	39 (5.9)	-	Not described
Uslu & Adıgüzel Akbaba, 2024 Turkey	Cross-sectional	General, cardiothoracic, urogenital, neurosurgery and other unspecific	Not described	240	45 (18.8)	-	Not described
Usul & Dizer, 2025 Turkey	Cross-sectional	Ear-nose-and throat, neuro, general, thoracic, urological, and orthopaedic surgery	Supine, lateral, prone and lithotomy	140	36 (25.7)	-	Directly after surgery and 24 h after surgery
Wright et al., 2014 Australia	Cross-sectional	Head and neck surgery	Not described	88	13 (14.0)	-	Not described
Wu et al., 2021 China	Retrospective cohort	Neurosurgery	Supine, prone, and lateral	465	69 (14.8)	-	Not described
Yılmaz & Başlı, 2021 Turkey	Cross-sectional	Orthopaedic, neuro, general, urological, plastic, obstetrics, gynaecological, and ear, nose and throat surgery	Supine, prone, lateral, and lithotomy	164	11 (6.4)	-	Directly after surgery and 24 h after surgery
Yoshimura et al., 2015 Japan	Cross-sectional	Not specified	Park-Bench position	29	7 (24.1)	-	30 min after surgery, followed by review of medical records for one week
*Peripheral nerve injuries*							
Behera et al., 2025 India	Cross-sectional	General, orthopaedic, gynecological, neuro, and urological surgery	Supine, lateral, prone, and lithotomy	110	7 (6.4)	Ulnar Brachial Plexus Radial	6, 24 and 48 h after surgery
Holtzman et al., 2017 USA	Retrospective cohort	Shoulder surgery	Beach chair	397	5 (1.3)	Lateral femoral cutaneous	At first postoperative visit
Koç et al., 2012 USA	Retrospective cohort	Robot-assisted laparoscopic prostatectomy	Trendelenburg with split-leg	377	5 (1.3)	Obturator Femoral	Within 24 h, during the first 7 days and up to 1 year after surgery
Navarro-Vicente et al., 2012 USA	Prospective cohort	Colorectal surgery	Supine or Lloyd-Davis	2 304	8 (0.3)	Ulnar Musculocutaneous Axillary Brachial Plexus Tibial Interosseous Lateral popliteal	Not described
Posta et al., 1997 USA	Retrospective cohort	Hip arthroplasty	Not described	7 150	16 (0.2)	Ulnar Median Axillary Brachial	Up to 2 years after onset of injury
Warner et al., 1994a USA	Case- control 1:3	Not described.	Lithotomy position	198 461	55 (0.028)	Connon peroneal Sciatic Femoral	At least 3 months after surgery up to 5 years
Warner et al., 1994b USA	Case-control 1:3	Diagnostic, noncardiac procedures	Supine, prone, lithotomy, lateral, other (not described)	1 129 692	414 (0.037)	Ulnar	At least 3 months after surgery
Zarandona del Campo et al., 2023 Spain	Prospective cohort	Pelvic laparoscopy in general, urological and gynecological surgery	Trendelenburg	101	13 (12.9)	Not described specifically	48 h postoperatively and 1 month after surgery

Notes: Injury-specific outcomes apply only to studies on peripheral nerve injuries

A total of 26 074 patients were enrolled in the studies on pressure injuries, of whom 2 001 developed an intraoperatively acquired pressure injury (category I or II). This resulted in an overall incidence of 6.6% with a range of 1.8% (Peng et al., 2024) to 41.8% (Dong et al., 2021). Sample sizes varied from 29 (Yoshimura et al., 2015) to 11 231 (Chen et al., 2024). The studies were conducted across multiple surgical contexts with cardiovascular surgery most frequently represented (n = 11). Supine-, prone-, and lateral positions were the most commonly reported. Follow-up ranged from immediately after surgery to 10 days postoperatively, and 12 studies did not specify follow-up timing.

The 8 studies examining peripheral nerve injuries showed greater variability in methodological approaches: 3 retrospective studies, 1 cross-sectional study, 2 case-control studies, and 2 prospective studies. In total, 1 338 592 patients were enrolled, with individual cohorts ranging from 101 (Zarandona Del Campo et al., 2023) to 1 129 692 (Warner et al., 1994b). Reported incidence ranged from 0.0003% (Warner et al., 1994b) to 12.9 % (Zarandona Del Campo et al., 2023). The studies covered several surgical specialties and patient positions. Follow-up intervals ranged from within the first 24 h (Koç et al., 2012; Behera et al., 2025) to five years after surgery (Warner et al., 1994a). Most of the included studies (n = 5) examined peripheral nerve injuries affecting predefined peripheral nerves in the upper and/or lower extremities.

### 3.3 Risk of bias in studies

Among the included studies on pressure injuries, 15/33 studies were assessed as having a high overall risk of bias, while 7/8 studies on peripheral nerve injuries were rated high overall risk of bias. Across domains, performance and reporting bias were the most frequent concerns, with detection bias also commonly assessed high or unclear. Selection bias items were often not applicable to the included designs. The most common potential sources of bias related to incomplete reporting of outcome assessment, including timing of injury assessment, surgical position and follow-up. Item- level assessments are detailed in Supplementary Table S1.

### 3.4 Risk factors for pressure injuries

Intrinsic and extrinsic variables examined as potential risk factors for pressure injuries and reported in three or more studies are summarised in Table 2. A total of 101 variables were assessed across the included studies. Among intrinsic risk factors, sex, age, and body mass index (BMI) were the variables most frequently analysed, while surgery duration was the most consistently examined extrinsic risk factor.

**Table 2:** Pre- and intraoperative risk factors for pressure injuries.

Sex was the most frequently studied variable, investigated in 25 of the 33 included studies. However, only two studies reported statistically significant findings, both identifying male sex as a risk factor for pressure injury (Yoshimura et al., 2015; Wu et al., 2021).

Age was the second most investigated intrinsic variable and was examined in 21 studies. Of these, 11 found increasing age to be a statistically significant risk factor. However, one study reported the opposite pattern, with younger patients more likely to sustain a pressure injury (Wright et al., 2014). Among studies using categorical age groups (Buso et al., 2021; İlkhan and Sucu Dag, 2023; Luo et al., 2019; Ramezanpour et al., 2018; Shi et al., 2023; Usul and Dizer, 2025), considerable variation in threshold definitions precluded pooling. One study included only participants aged 65 years and older (Uslu and Adıgüzel Akbaba, 2024).

BMI was investigated in 22 studies. The reported findings varied according to how BMI was categorised and analysed across studies. Some studies analysed BMI as a continuous variable, whereas others used categorical BMI groups with different cut-off values. A significantly higher BMI among patients with pressure injuries were found in 8 studies (Forni et al., 2021; Wu et al., 2021; İlkhan and Sucu Dag, 2023; Karahan et al., 2022; Peng et al., 2024; Shi et al., 2023; Ninlaphut et al., 2025; Li et al., 2025), whereas a significantly lower BMI among patients was identified in 4 studies (Luo et al., 2019; Karahan et al., 2022; Shi et al., 2023; Suh et al., 2021). Karahan et al. (2022) and Shi et al. (2023) reported significant findings for both lower and higher BMI categories. Other intrinsic factors, such as diabetes, albumin, haemoglobin, and various comorbidities, were analysed less frequently and showed inconsistent findings (Table 2).

Among the investigated extrinsic factors, surgery duration was the most frequently studied, being examined in 28 studies (Table 2). In the narrative synthesis of the individual studies, 21 of the 28 studies reported significantly longer surgery duration or a statistically significant relationship between longer surgery duration and pressure injury occurrence. Several studies applied minimum duration thresholds for inclusion, most commonly ≥2 h (Bulfone et al., 2012; Celik et al., 2019; Chen et al., 2019; Chen et al., 2017; Lei et al., 2022; Karadede et al., 2025; Yılmaz and Başlı, 2021) or ≥5 h (Wright et al., 2014). The intraoperative use of circulatory agents, such as vasopressors or inotropic drugs, was also associated with an increased incidence of pressure injuries. Other extrinsic variables, such as intraoperative blood loss and surgical positioning were reported less consistently. Surgical position varied by specialty, but in studies comparing positions, the prone and lateral positions were associated with higher risk than supine (Chen et al., 2024; Karahan et al., 2022; Peng et al., 2024; Shaw et al., 2014; Shi et al., 2023; Wu et al., 2021; Yılmaz and Başlı, 2021).

### 3.5 Risk factors for peripheral nerve injuries

Compared with pressure injuries, the evidence on peripheral nerve injuries was considerably smaller in volume, with fewer studies available for analysis. In total, 27 intrinsic and extrinsic variables were examined (Table 3), and most were assessed in only one or two studies, limiting the possibility of identifying consistent patterns across the literature.

**Table 3:** Intrinsic and extrinsic risk factors for peripheral nerve injuries.

Among intrinsic factors, age and BMI were the variables most frequently associated with peripheral nerve injuries. Three studies reported that increasing age was significantly related to a higher risk of peripheral nerve injury (Warner et al., 1994a; Warner et al., 1994b; Zarandona Del Campo et al., 2023). BMI was statistically significant in different directions across studies: higher BMI in one study (Holtzman et al., 2017), low BMI ≤ 20 kg/m² in another (Warner et al., 1994a), and both low (<24 kg/m²) and high (>38 kg/m²) in a third (Warner et al., 1994b), making data pooling precluded due to differing cutoff definitions. Sex was analysed in six studies but reached statistical significance in only one (Warner et al., 1994b).

Among the extrinsic risk factors, surgery duration was the only variable consistently identified as statistically significant, reported in three studies (Koç et al., 2012; Warner et al., 1994a; Zarandona Del Campo et al., 2023). In the study by Warner et al. (1994a), patients positioned in lithotomy position for procedures exceeding 4 hours demonstrated a higher incidence of peripheral nerve injury, while Zarandona Del Campo et al. (2023) reported that affected patients had a longer median surgery duration and further showed that prolonged time in the Trendelenburg position was associated with an increased risk of peripheral nerve injury (p = 0.011). Other intrinsic and extrinsic factors were examined less frequently or produced inconsistent or non-comparable results.

### 3.6 Meta-analyses of risk factors for pressure injuries

There were nine risk factors for pressure injury selected for meta-analysis: sex, age, BMI, diabetes, hypertension, lower preoperative haemoglobin, lower preoperative serum albumin, surgery duration, and intraoperative use of circulatory agents (Supplementary Table S2). The sample size ranged from 29 (Yoshimura et al., 2015) to 3 840 (Luo et al., 2019) across studies.

**Fig. 2:**
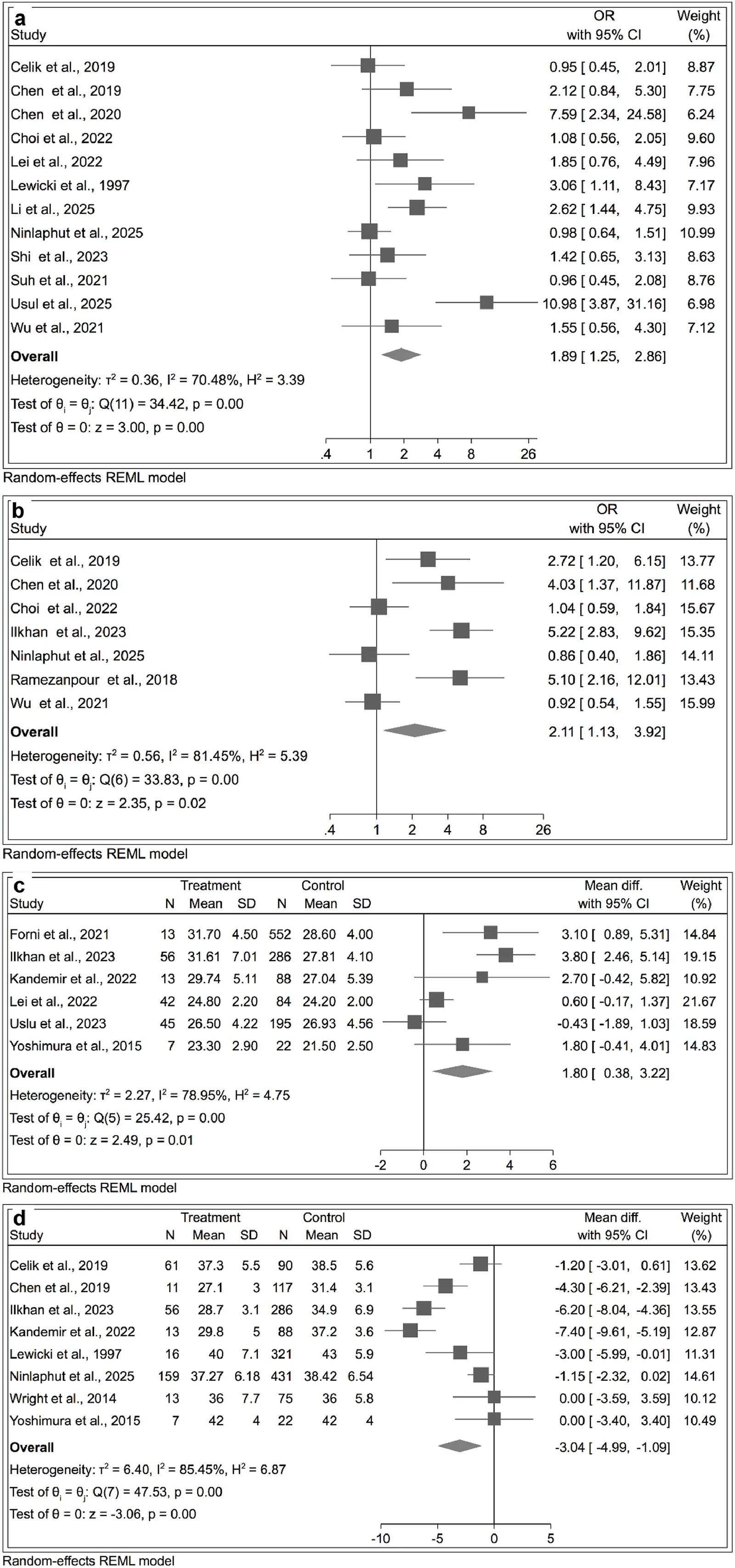
Forest plots of selected risk factors for pressure injuries. a) Diabetes; b) Intraoperative use of circulatory agents; c) Body Mass Index; d) Preoperative serum albumin. Effect estimates are presented as Odds Ratios (ORs) or Mean Differences (MDs) with 95% confidence intervals. Random-effects models were used in all analyses.

In random-effects meta-analyses several risk factors showed statistically significant pooled effects. Among the dichotomous variables, diabetes (OR 1.89; 95% CI 1.25–2.86; p < 0.001; I² = 70.48%) and intraoperative use of circulatory agents (OR 2.11; 95% CI 1.13–3.92; p = 0.02; I² = 81.45%) were associated with increased odds of pressure injury. Among continuous variables, patients who developed pressure injuries had a higher BMI (MD 1.80 kg/m²; 95% CI 0.38 to 3.22; p = 0.01; I² = 78.95%) and lower preoperative serum albumin levels (MD -3.04 g/L; 95% CI -4.99 to -1.09; p < 0.001; I² = 85.45%) compared with those without pressure injuries. Selected pooled estimates are presented in Fig. 2.

In contrast, sex (OR 1.10; 95% CI 0.94–1.30; p = 0.23; I² = 44.11%), age (MD 0.69 years; 95% CI -1.65 to 3.03; p = 0.56; I² = 69.35%), hypertension (OR 1.37; 95% CI 0.93–2.01; p = 0.11; I² = 61.84%), preoperative haemoglobin (MD -7.64 g/L; 95% CI -16.90 to 1.62; p = 0.11; I² = 90.27), and surgery duration (MD 42.54 minutes; 95% CI -9.03 to 94.11; p = 0.11; I²= 98.68%) did not show statistically significant pooled differences (Supplementary Table 2). Overall, between-study heterogeneity ranged from moderate (sex) to considerable (surgery duration and preoperative haemoglobin).

### 3.7 Sensitivity and influence analyses

Sensitivity and influence analyses were conducted for the pressure injury meta-analyses. Sensitivity analyses (Supplementary Figures S1-S9) restricting the data to studies with low risk of bias or to cross-sectional designs yielded effect sizes that were consistent with the main analyses, with only minor changes in pooled estimates. Excluding studies with high risk of bias slightly altered the significance for two variables: BMI was no longer statistically significant (MD 1.17 kg/m²; 95% CI - 0.41 to 2.76), whereas preoperative haemoglobin reached significance (MD -9.05 g/L; 95% CI -16.82 to -1.29). When retrospective and case-control studies were excluded, the pooled effects for sex (OR 1.14; 95% CI 0.92– 1.41) and diabetes (OR 2.05; 95% CI 0.88– 4.80) were no longer statistically significant. Results for all other analysed risk factors remained robust. Additional sensitivity analyses restricted to crude estimates were performed for diabetes, hypertension and intraoperative use of circulatory agents (Supplementary Figures S1-S9). The pooled effect estimates for diabetes (OR 2.07; 95% CI 1.31-3.27) and intraoperative use of circulatory agents (OR 2.41; 95% CI 1.40-4.17) remained statistically significant and were consistent with the primary analyses. For hypertension, the pooled effect estimate remained similar in magnitude but became statistically significant (OR 1.60; 95% CI 1.14-2.25).

Influence analyses were conducted, and overall, removal of individual studies did not materially affect the pooled estimates, suggesting that the results were not driven by any single study (Supplementary Figures S10-S18).

### 3.8 Publication bias

Publication bias was assessed for the meta-analyses for pressure injury risk factors. Publication bias was assessed using funnel plots (Supplementary Figures S19-S21) and Egger’s regression tests for the variables sex, diabetes, and age. Visual inspection revealed clear asymmetry for diabetes and some degree of asymmetry for age, whereas the plot for sex appeared more symmetrical. Egger’s regression test indicated evidence of small-study effects for diabetes (p = 0.006), supporting the visual impression, while no statistically significant evidence of such effects was observed for sex (p = 0.068) or age (p = 0.604).

Overall confidence in the evidence was considered lower for diabetes due to small-study effects and substantial heterogeneity, whereas confidence for sex and age was assessed as moderate given more consistent findings and no statistically significant evidence of small-study effects.

## 4 Discussion

This systematic review and meta-analysis aimed to identify and synthesise intrinsic and extrinsic risk factors for position-related pressure injuries and peripheral nerve injuries. The meta-analyses revealed statistically significant associations between pressure injury and the intrinsic risk factors: diabetes, higher BMI and reduced preoperative serum albumin levels. Among the extrinsic risk factors, the use of circulatory agents was identified as a statistically significant contributor.

For peripheral nerve injuries, the absence of meta-analyses reflects the limited and heterogeneous reporting of potential risk factors across studies. In addition, no individual factor met the predefined threshold of more than five studies required to justify pooling using REML, which restricts the ability to draw quantitative conclusions. The lack of evidence on position-related peripheral nerve injuries can be explained by the complexity of the aetiology and sometimes late appearance (Welch et al., 2009). Since the development of a peripheral nerve injury is generally multifactorial, it may be challenging to determine whether it has been caused by incorrect or prolonged positioning or by other factors (Menezes et al., 2013). This makes a peripheral nerve injury much more difficult to prevent.

For both pressure injuries and peripheral nerve injuries, descriptive synthesis of the reported risk factors was challenging due to heterogeneity in the variables assessed and inconsistent reporting across studies. One study (Menezes et al., 2013) addressed both pressure injuries and peripheral nerve injuries within the same investigation.

Intraoperatively acquired position-related injuries are some of the implicit risks of surgery and it is necessary for perioperative nurses and other healthcare professionals to have knowledge of current risk factors to do accurate evaluations (Özdemir et al., 2023). Patient advocacy is even more important in a high-technological environment as the operating room, where the patients need continuous assistance with their vital functions. Gathering, sharing, and analysing information about the patients’ anamnesis and experiences, are fundamental for risk assessments and providing targeted preventive measures during the perioperative care (Sundqvist et al., 2018), why the results in this review are important in perioperative nursing.

Surgical duration was the most reported statistically significant risk factor in individual studies on pressure injuries. However, the meta-analysis did not demonstrate a statistically significant pooled effect, in contrast to Haisley et al. (2020). This discrepancy may reflect the limited number of studies eligible for meta-analysis and methodological heterogeneity, including differences in minimum surgery duration thresholds and postoperative skin assessment.

Results from previous systematic reviews by Haisley et al. (2020) and Nasiri et al. (2021) verify our results in their meta-analyses regarding diabetes as a risk factor, significantly associated with pressure injury (RR 1.49, OR 1.985, respectively OR 1.52). According to Nasiri et al. (2021), diabetes may be the most significant risk factor for pressure injury, as it can impair blood flow within epidermal layers and thereby damage the underlying vascular structure. Glycaemic control should be assessed frequently prior to surgery, either in primary care or during the pre-anaesthesia consultation, to ensure optimal diabetes management (Stewart and Selzer, 2022).

Intraoperative hypotensive episodes and the use of circulatory agents are closely related. While hypotension, particularly episodes of intraoperative low diastolic blood pressure (<60 mm Hg), was examined in seven of the included studies, the data were not suitable for pooling. A meta-analysis was, however, conducted for the intraoperative use of circulatory agents, which are administered to counteract hypotension during surgery. Although vasoconstriction induced by circulatory agents may reduce perfusion in the skin and other peripheral tissues, these agents are often lifesaving by restoring systemic blood pressure and maintaining perfusion of vital organs. As intraoperative hypotension and the use of circulatory agents are often difficult to avoid, management strategies need to be tailored to the underlying cause. When circulatory agents are required, their administration should serve as an early warning for perioperative nurses to intensify pressure injury prevention strategies (Kebapci and Tilki, 2024).

Our findings are consistent with previous reports demonstrating an association between low preoperative serum albumin and pressure injury development (Kim et al., 2018). However, this association should be interpreted with caution, as hypoalbuminaemia may reflect underlying frailty, systemic inflammation, malnutrition or overall disease burden rather than representing an independent causal risk factor for pressure injury development (Allison and Lobo, 2024). Consequently, serum albumin may be better regarded as a marker of underlying clinical vulnerability than an independent risk factor.

The patients included in the individual studies underwent surgery in different positions. There were difficulties comparing the association of injuries and surgical positions due to diversities in reference variables and inclusion criteria. The most common surgical position is the supine position (Gefen et al., 2020). In this review, the prone and lateral positions were associated with pressure injuries to a greater extent than the supine or lithotomy positions. Regardless of the intraoperative position, remaining immobilised for a prolonged period increases the risk of position-related injuries due to pressure mechanisms, friction and shearing forces (Menezes et al., 2013). Strategies such as avoiding overstretching of the extremities, ensuring adequate padding, and minimising pressure are recommended to reduce the risk of perioperative neuropathies (American Society of Anesthesiologists Task Force on Prevention of Perioperative Peripheral Neuropathies, 2018).

Knowledge of individual risk factors for position-related injuries is the first element in a bundle of preventive strategies to avoid these injuries. Prehabilitation is another important concept to improve the physiological, metabolic, and psychological resilience before surgery to enhance functional capacity. It is important to establish an optimal baseline for the patient involving a multidisciplinary approach. Modifiable risk factors like smoking, malnutrition, poor physical activity, and low psychological preparedness can be optimised before surgery to avoid perioperative stressors (West et al., 2021). Another approach that could improve recovery after surgery is the ERAS concept (Enhanced Recovery After Surgery) which consists of other perioperative elements for patients in different surgical settings, such as medical optimisation of chronic diseases, preoperative nutritional support, preoperative carbohydrate treatment, early mobilisation after surgery, to mention a few (Gustafsson et al., 2025).

The surgical field is in constantly changing with new surgical methods, such as minimally invasive and robotic surgery that may demand advanced surgical positions (Mills et al., 2013, Al-Temimi et al., 2017). At the same time, society is also facing an increasingly older surgical population with various comorbidities, presenting challenges due to the physiological changes associated with aging. To reduce the risk of postoperative complications, accurate preoperative assessments are necessary to ensure surgical safety (Kolarsick et al., 2020). Post-discharge follow-up is also important to identify complications that require documentation and, where necessary, treatment (Martins et al., 2023). Future research may focus on the effect of prehabilitation, together with intra- and postoperative nursing interventions, to avoid and/or decrease the severity level of position-related injuries. Individual follow-up, considering underlying conditions, age, and duration of surgery, should also be investigated to achieve the most favourable outcomes.

## 5 Limitations

The primary limitation in this systematic review was the heterogeneity of the included studies, encompassing both clinical and methodological variation. There were variations in the study population regarding surgical specialty, urgency of surgery, surgery duration, and surgical positions, but also methodological differences in study design and high risk of bias in several studies. This indicate that the pooled estimates for some risk factors shall be interpreted cautiously. Many studies were of lower methodological quality because of their retrospective design. However, sensitivity analyses restricted to studies with lower risk of bias yielded comparable results. For some risk factors, adjusted estimates were not available for every study, and therefore crude and adjusted estimates were combined in the primary meta-analyses. However, a sensitivity analysis using only crude estimates was performed to assess the impact of this and generally yielded comparable results. Variables analysed as nominal or ordinal data, were categorised differently across studies, making direct comparisons difficult. This reduced the number of studies that could be included in the meta-analyses and limited the synthesis and interpretation of the results.

Another limitation was that pressure injuries and peripheral nerve injuries were searched within the same strategy. This approach resulted in the inclusion of older studies, particularly concerning pressure injuries, for which more recent literature exists. Nevertheless, all included studies fulfilled the predefined eligibility criteria.

Among the included studies there were systematic errors in measurements, such as performance and detection bias. The lack of information about when the assessments were performed and by whom, demonstrates weaknesses in the individual studies on both pressure and peripheral nerve injuries. In some studies, pressure injuries were assessed immediately after surgery, despite evidence that such injuries may not become clinically apparent until several days after surgery, increasing the risk of outcome misclassification. The individual studies on peripheral nerve injuries demonstrated comparable methodological shortcomings and were predominately evaluated as having a high risk of bias. Although risk of bias was systematically assessed at the individual study level, the overall certainty of evidence was not formally evaluated using a recognised certainty appraisal framework. Therefore, the findings should be interpreted with caution.

The restriction to studies published in English or Scandinavian languages may have introduced language bias. However, previous research suggests that language restrictions do not necessarily result in systematic bias when database searches are conducted comprehensively (Morrison et al., 2012).

This study also has several strengths. A thorough systematic literature search was conducted using multiple health databases. No further study was detected when hand-searching the reference lists, which suggests that the search strategy was comprehensive (Horsley et al., 2011). However, the possibility that some relevant or unpublished studies were missed cannot be entirely excluded. Authors of primary articles were contacted in case of ambiguities. Strict inclusion and exclusion criteria were established to reduce the risk of bias in the conduct of the review. Risk of bias in the included observational studies was assessed using the RTI item bank, which allows the assessment to be tailored to different observational study designs by omitting items that are not applicable (Viswanathan et al., 2013).

The geographical distribution of the included studies offers some variation across healthcare systems, which may support external validity. At the same time, differences in perioperative routines, preventive strategies, and reporting practices between regions, together with the more limited and concentrated evidence base for peripheral nerve injuries, indicate that generalisability should be interpreted with caution.

## 6 Conclusions

Surgical patients are at risk for position-related injuries due to a range of intrinsic and extrinsic factors. Meta-analyses have identified statistically significant associations between pressure injury and diabetes, high BMI, low preoperative serum albumin, and use of circulatory agents. These findings should, however, be interpreted with caution given the variability across studies. Further research into risk factors for peripheral nerve injuries is required to improve understanding of the complexity underlying of these conditions. Preoperative optimisation of patient health and thorough postoperative evaluations are essential for reducing these iatrogenic injuries.

## Funding

This research did not receive any specific grant from funding agencies in the public, commercial, or not-for-profit sectors.

## CRediT authorship contribution statement

**Veronica Ramirez Johansson:** Writing – review & editing, Writing – original draft, Visualization, Validation, Project administration, Formal analysis, Data curation, Conceptualization. **Oili Dahl:** Writing – review & editing, Validation, Conceptualization. **Ann-Charlotte Falk:** Writing – review & editing, Validation, Conceptualization. **Ulrica Nilsson:** Writing – review & editing, Validation. **Marina Dehara:** Writing – review & editing, Visualization, Validation, Software, Formal analysis. **Ann-Christin von Vogelsang:** Writing – review & editing, Visualization, Validation, Supervision, Resources, Funding acquisition, Formal analysis, Data curation, Conceptualization.

## Declaration of competing interest

The authors declare that they have no known competing financial interests or personal relationships that could have appeared to influence the work reported in this paper.

## Data Availability

Supplementary material is available online. All data supporting the findings of this study are available from the corresponding author upon reasonable request.
