# Supplementary material for: Risk factors for position-related injuries in adult surgical patients: a systematic review and meta-analysis
Source: Int J Nurs Stud Adv. 2026 Sep 8;11:100676. doi: 10.1016/j.ijnsa.2026.100676 (PMC13598170; doi:10.1016/j.ijnsa.2026.100676)
Supplement: Supplementary file 1 [file mmc4.pdf]

# Supplementary figures S1-S9 Sensitivity analyses

## Supplementary Figure S1. Sensitivity analyses for sex (male sex reference)

a) Excluding studies with high risk of bias

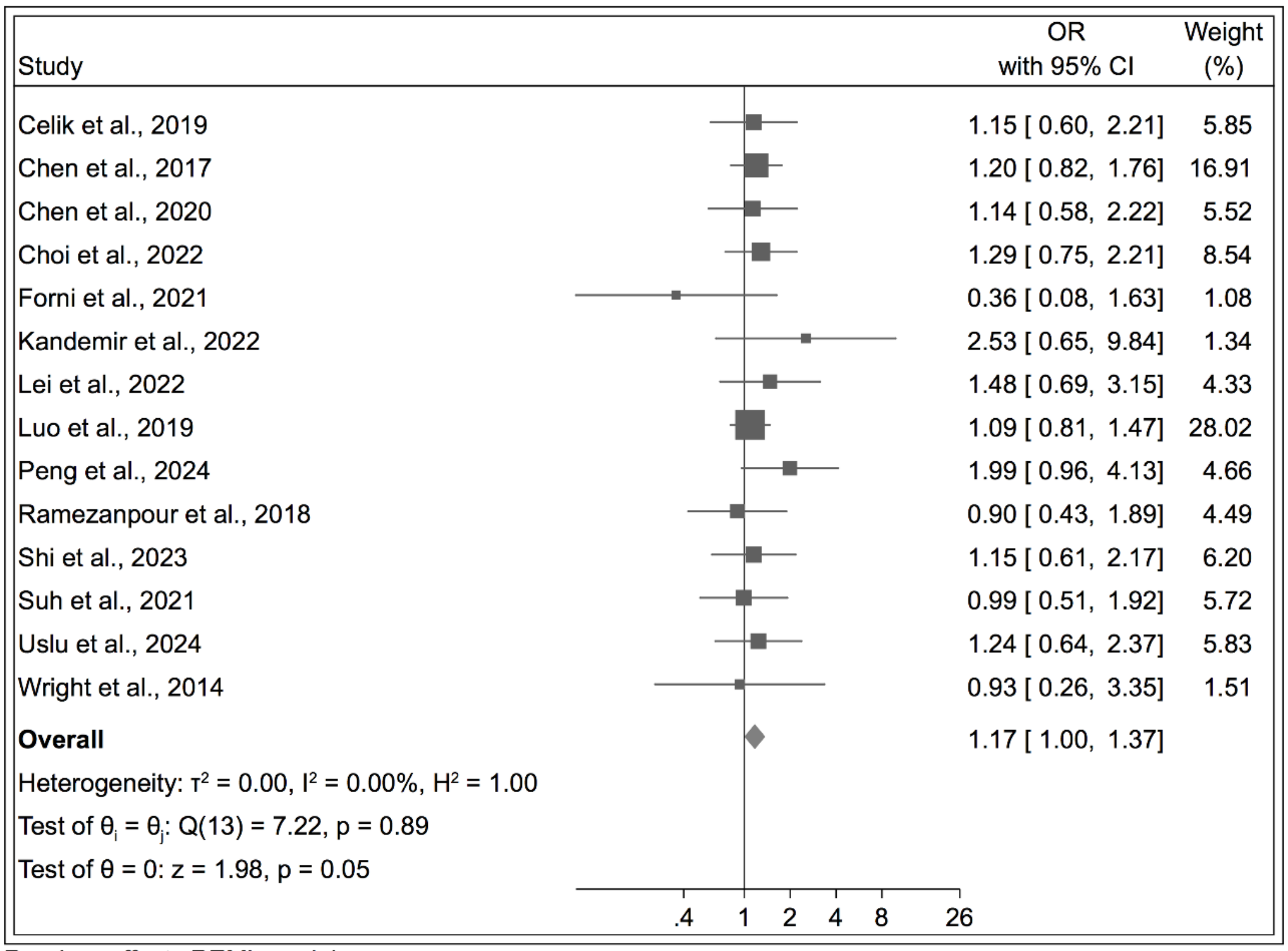


Random-effects REML model

b) Restricting analyses to cross-sectional studies

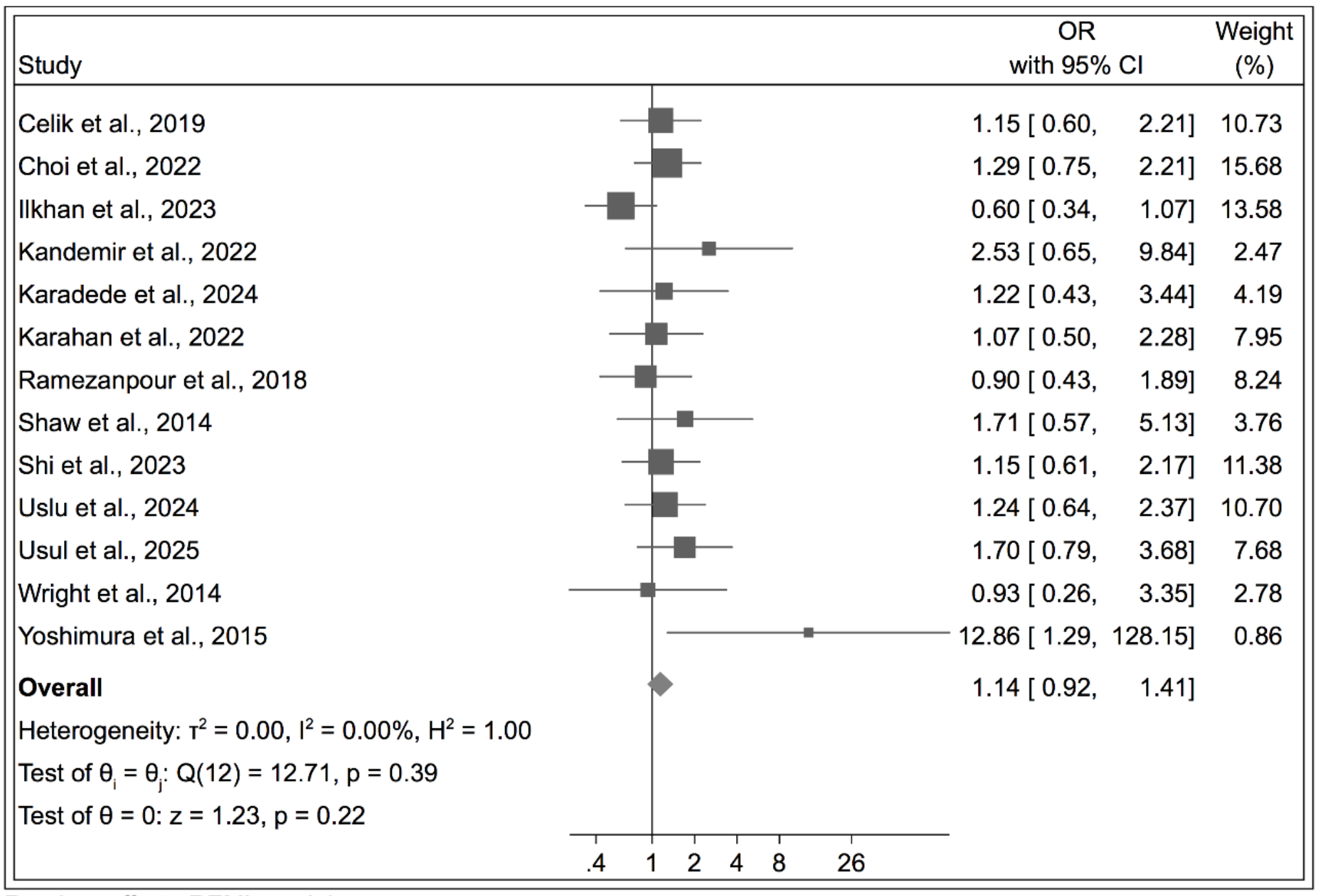


Random-effects REML model

**Supplementary Figure S2. Sensitivity analyses for diabetes**

a) Excluding studies with high risk of bias

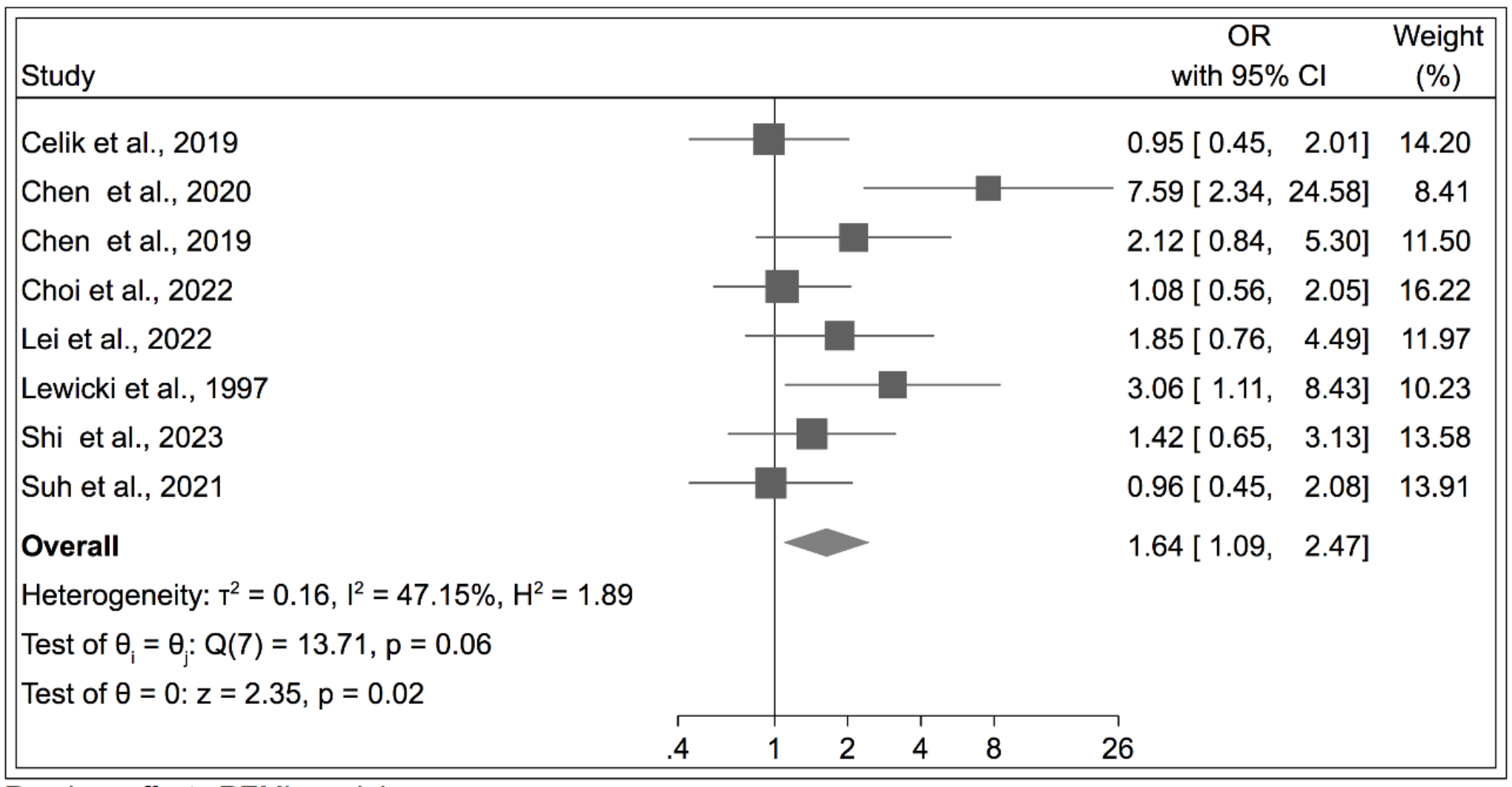


Random-effects REML model

b) Restricting analyses to cross-sectional studies

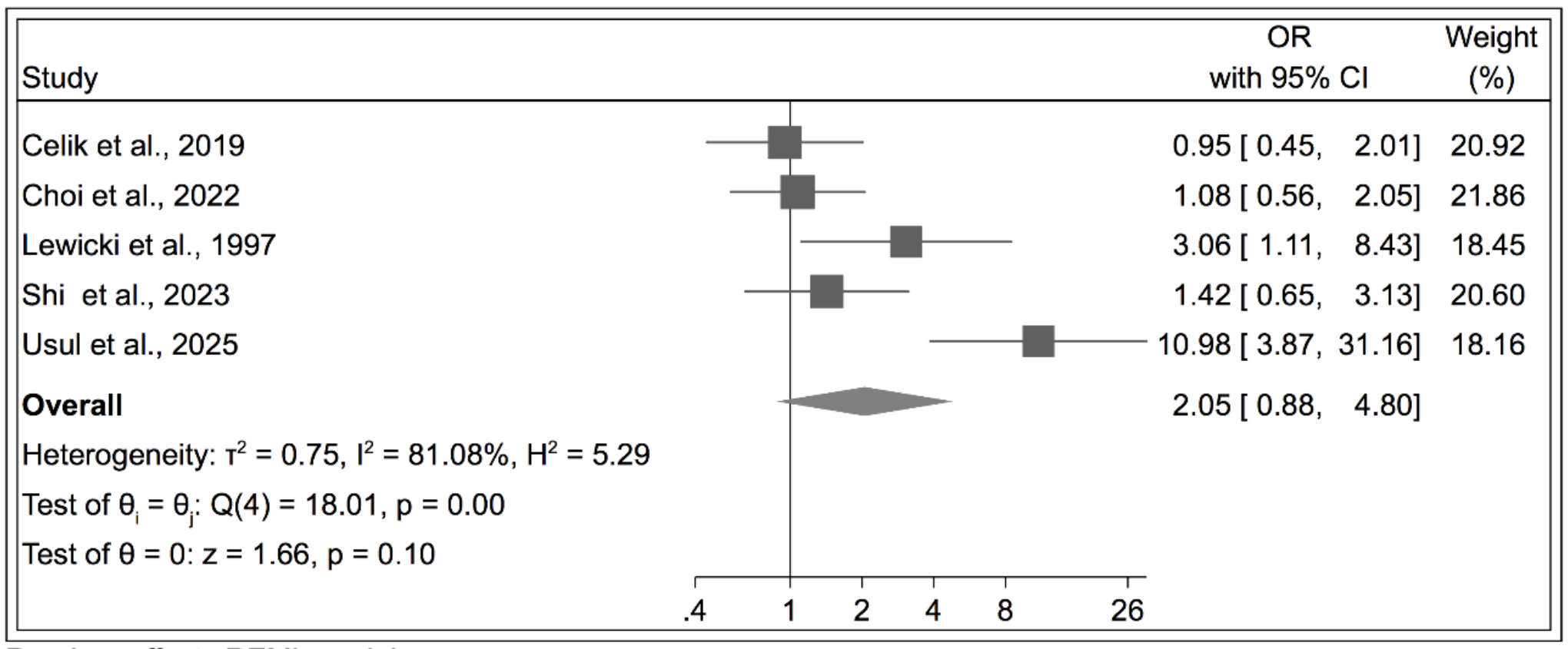


Random-effects REML model

c) Restricting analyses to crude effect estimates

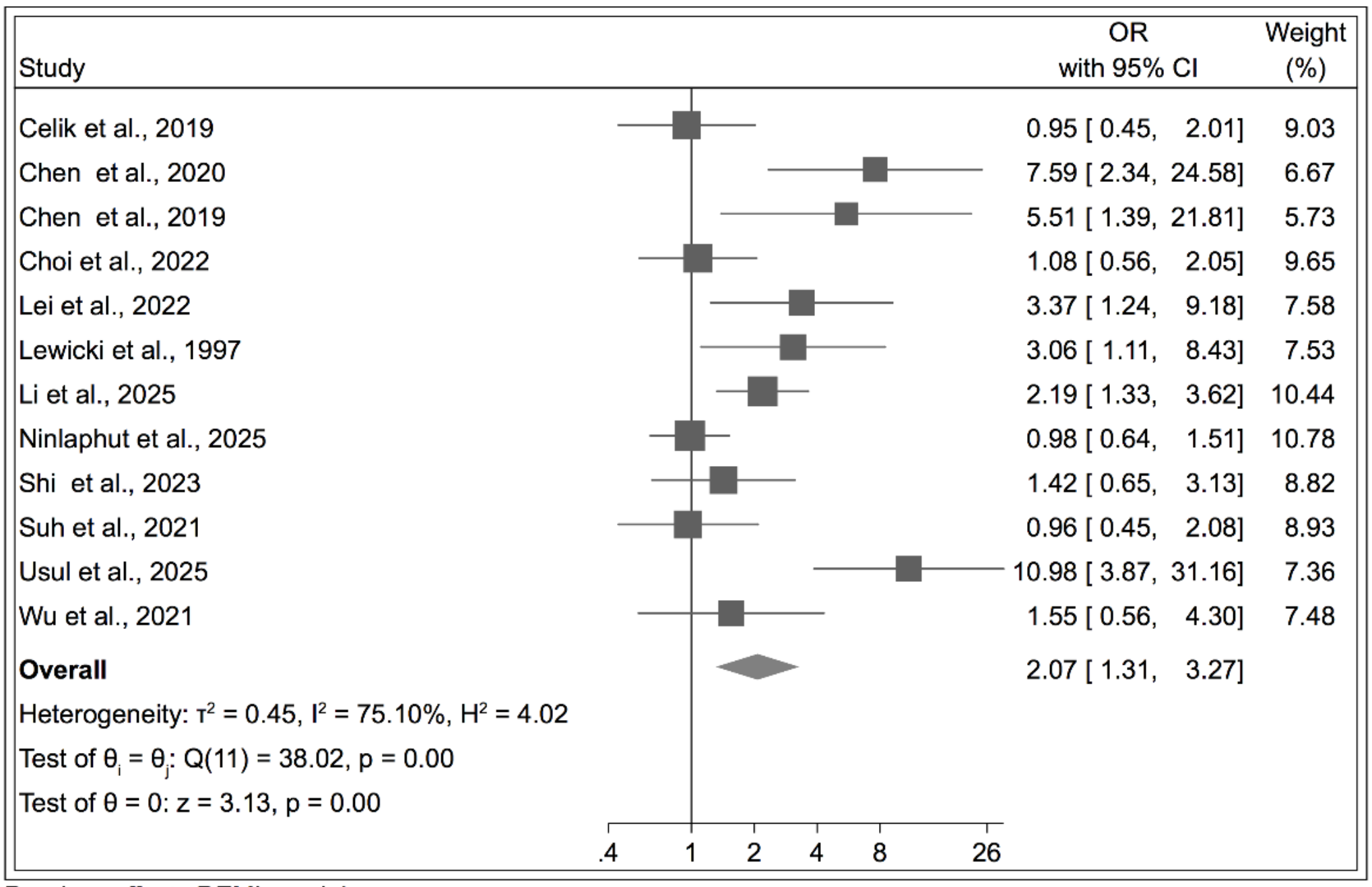


Random-effects REML model

**Supplementary Figure S3. Sensitivity analyses for hypertension**

a) Excluding studies with high risk of bias

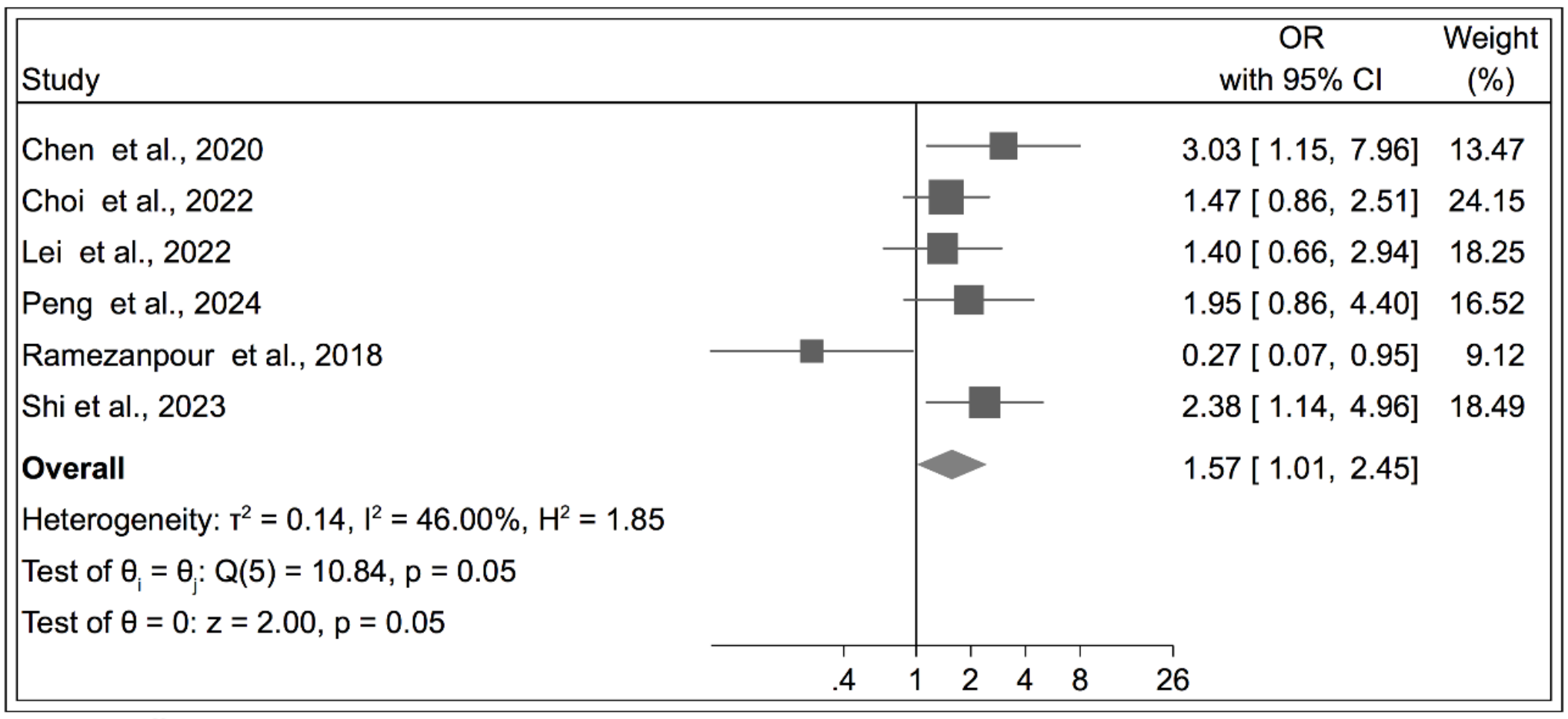

Random-effects REML model

b) Restricting analyses to cross-sectional studies

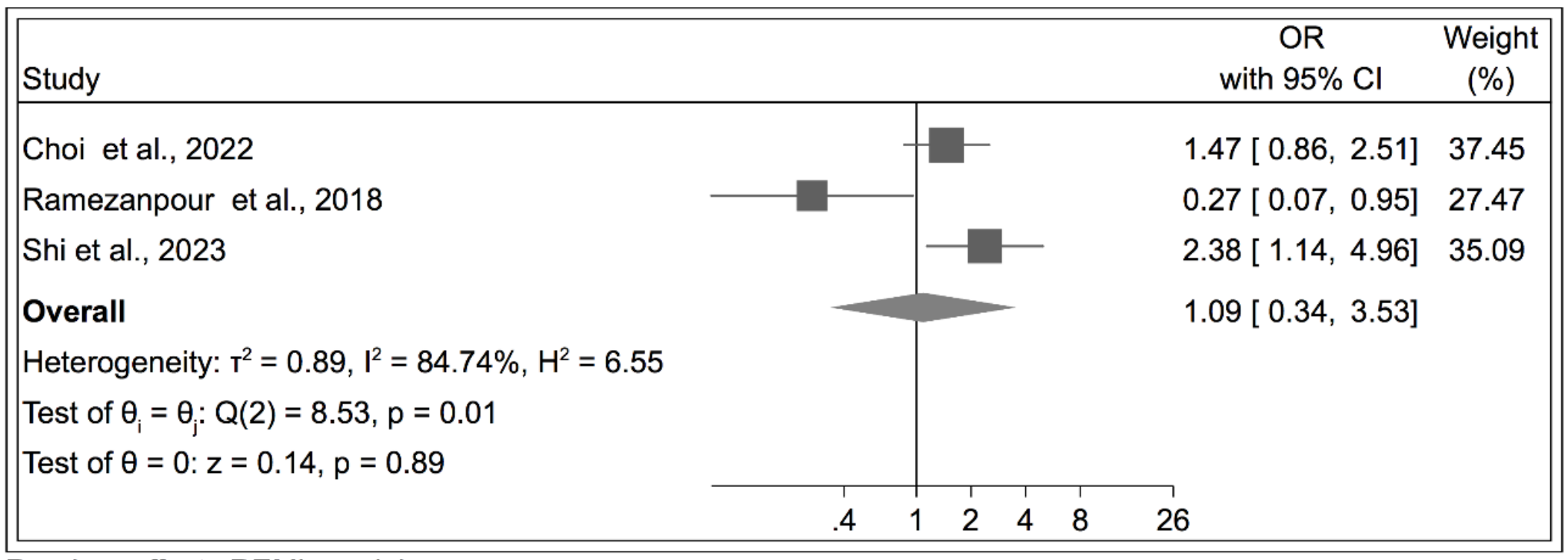

Random-effects REML model

c) Restricting analyses to crude effect estimates

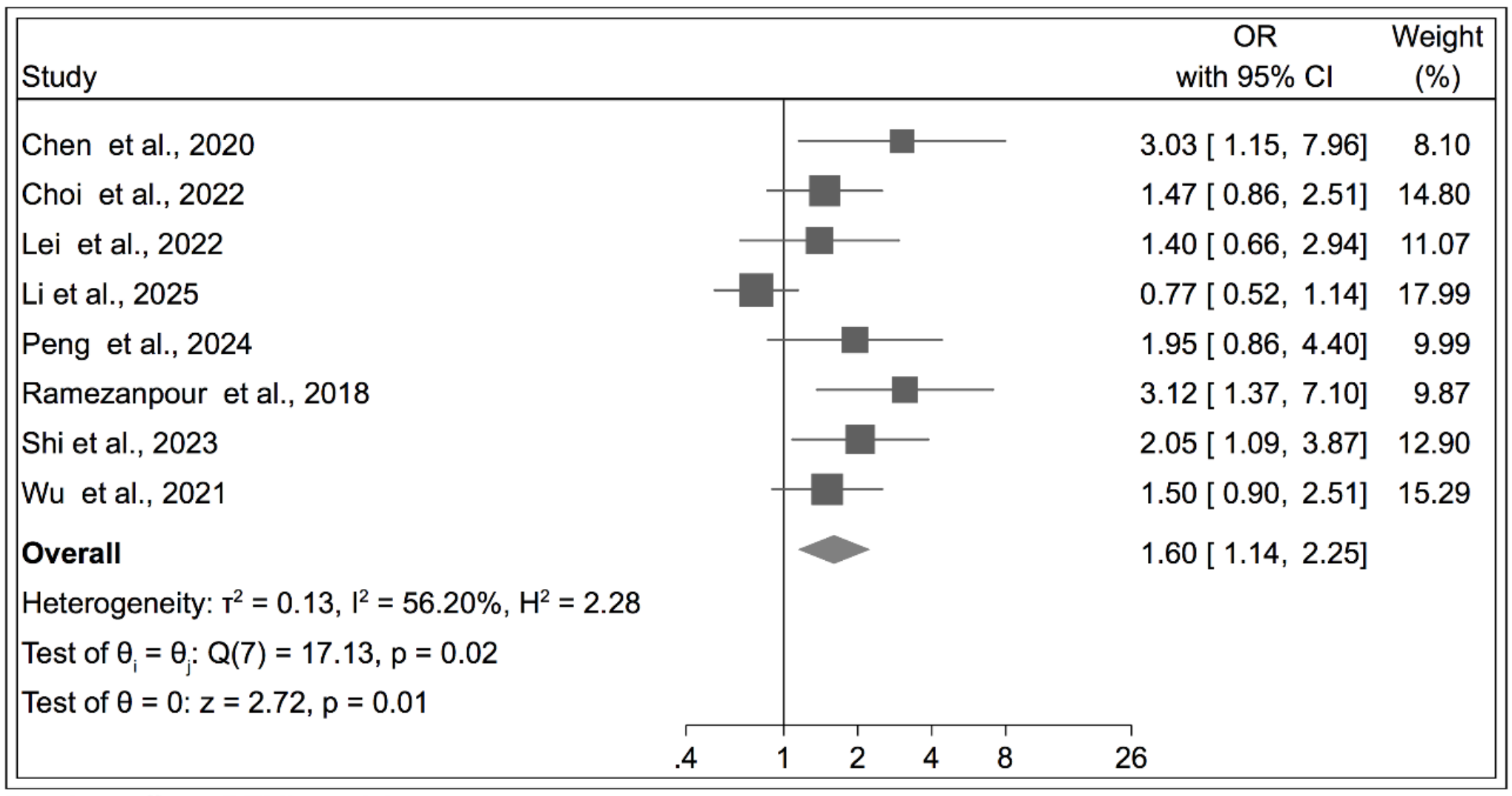

Random-effects REML model

## Supplementary Figure S4. Sensitivity analyses for age

a) Excluding studies with high risk of bias

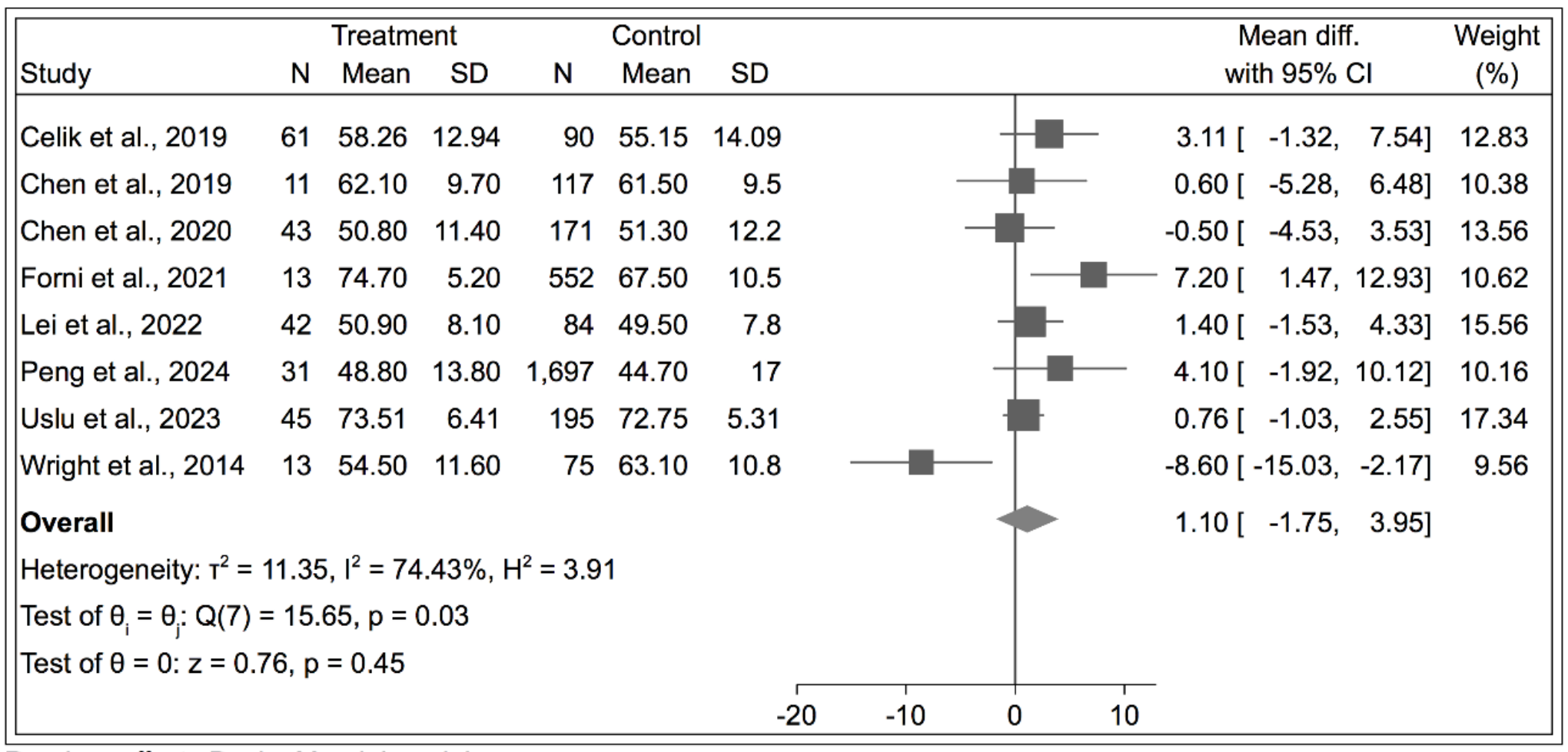


Random-effects Paule–Mandel model

b) Restricting analyses to cross-sectional studies

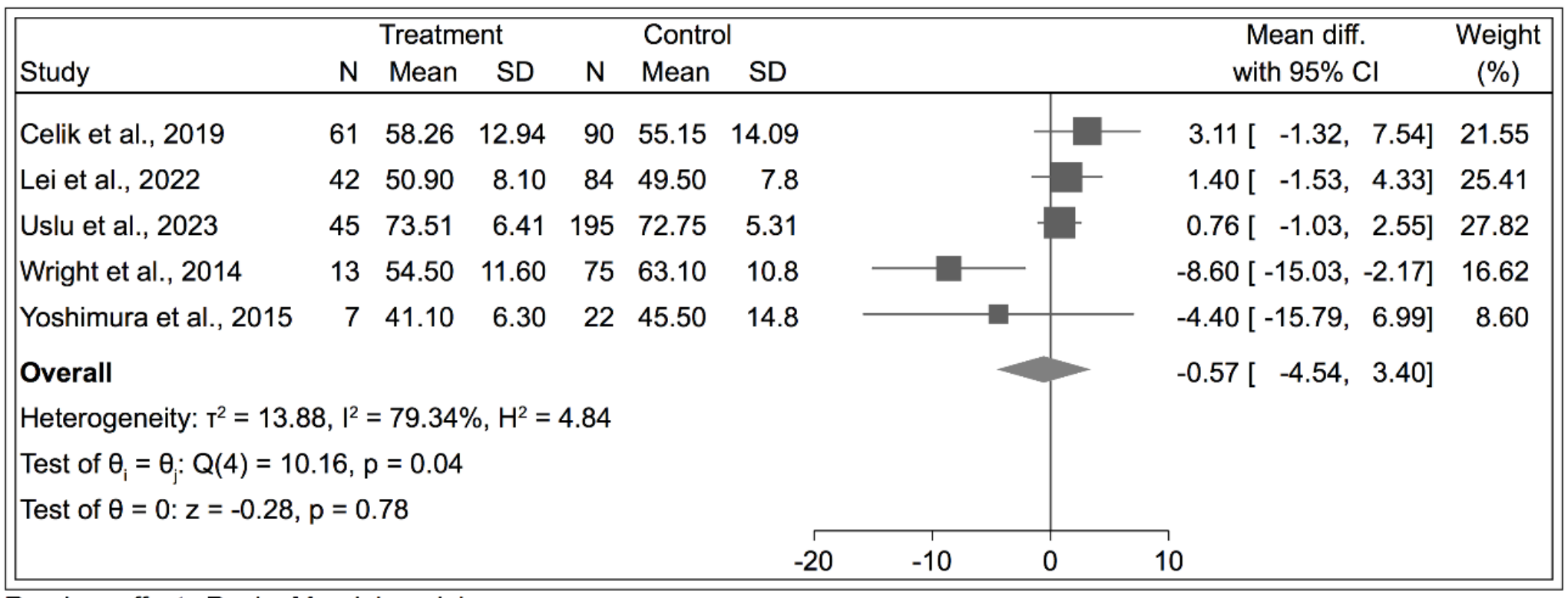


Random-effects Paule–Mandel model

## Supplementary Figure S5. Sensitivity analyses for Body Mass Index

a) Excluding studies with high risk of bias

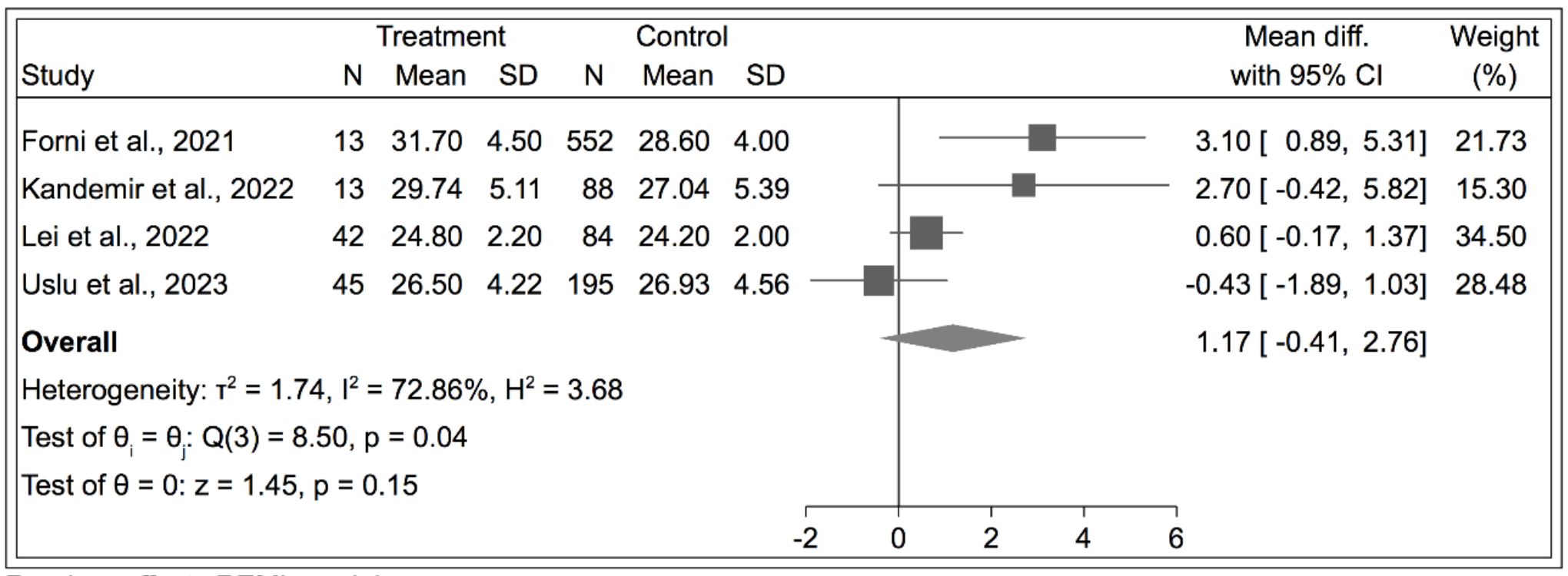


Random-effects REML model

b) Restricting analyses to cross-sectional studies

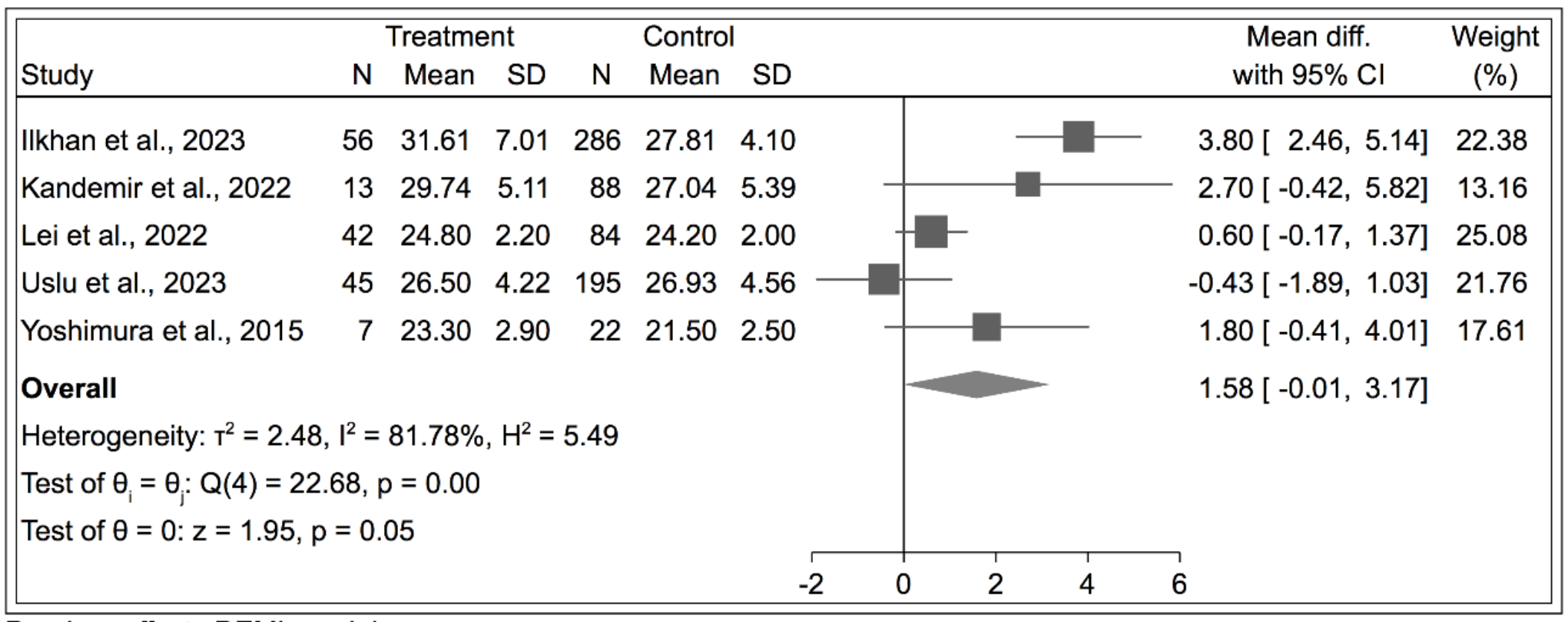


Random-effects REML model

## Supplementary Figure S6. Sensitivity analyses for preoperative albumin

a) Excluding studies with high risk of bias

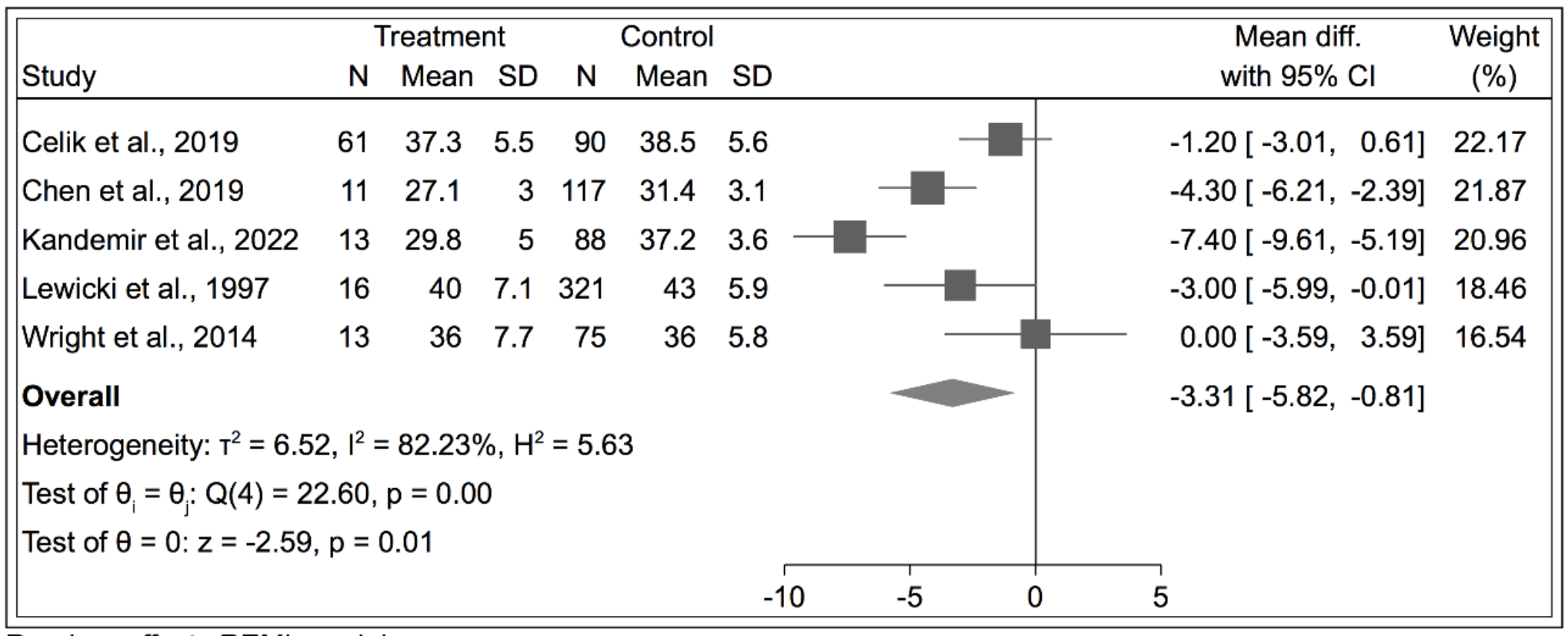


Random-effects REML model

b) Restricting analyses to cross-sectional studies

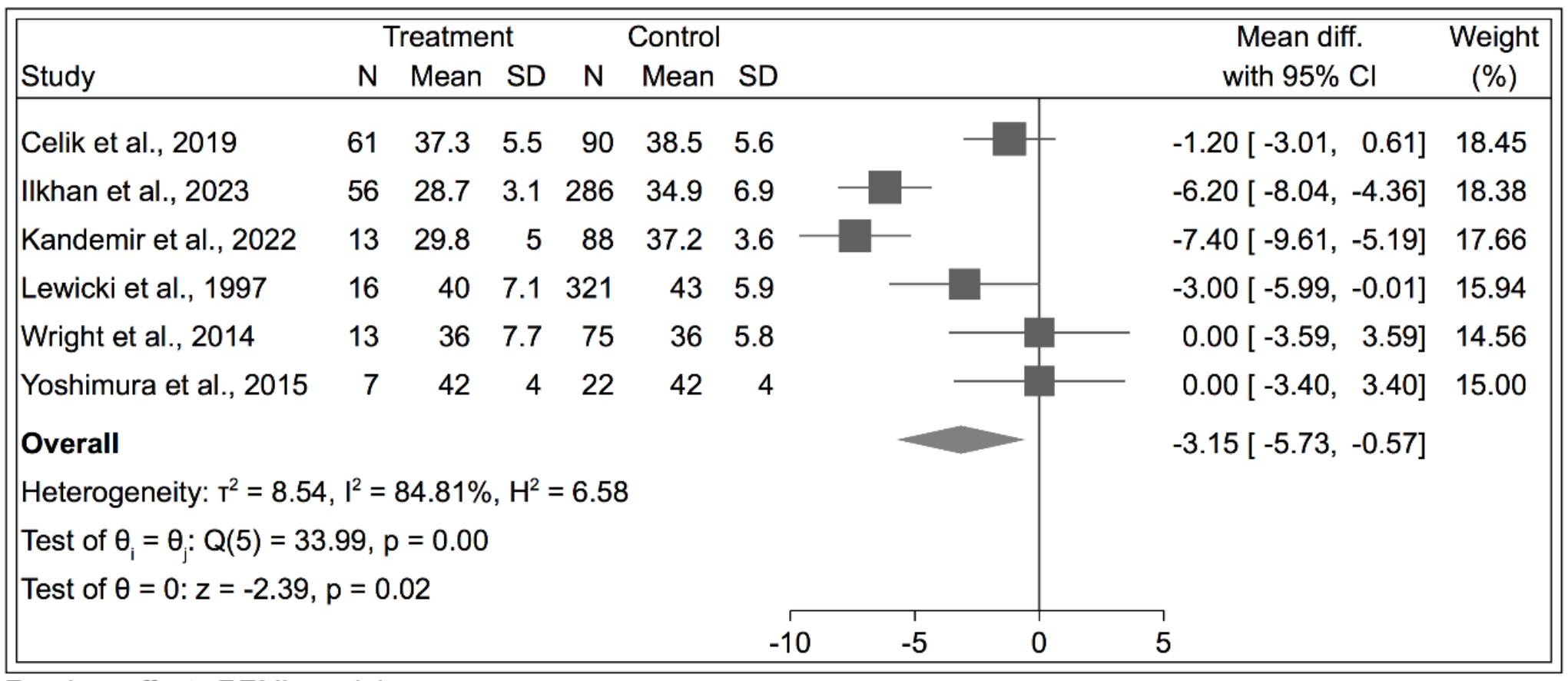


Random-effects REML model

## Supplementary Figure S7. Sensitivity analyses for preoperative haemoglobin

a) Excluding studies with high risk of bias

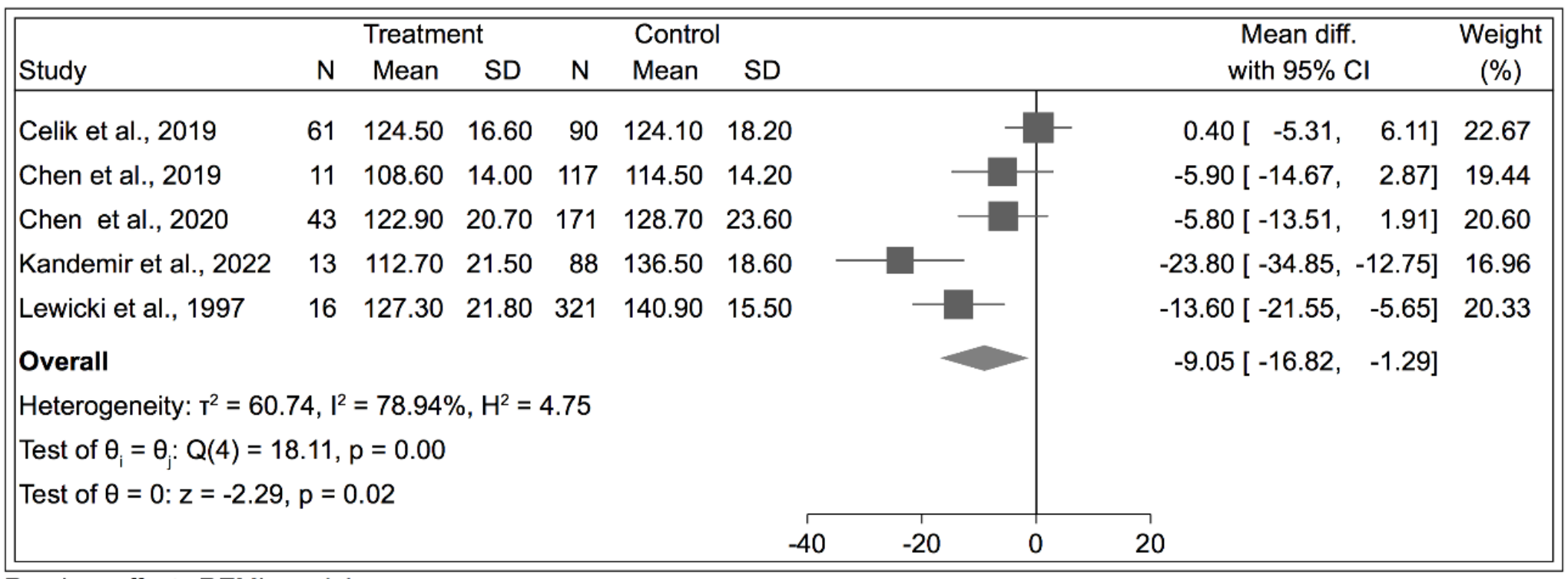


Random-effects REML model

b) Restricting analyses to cross-sectional studies

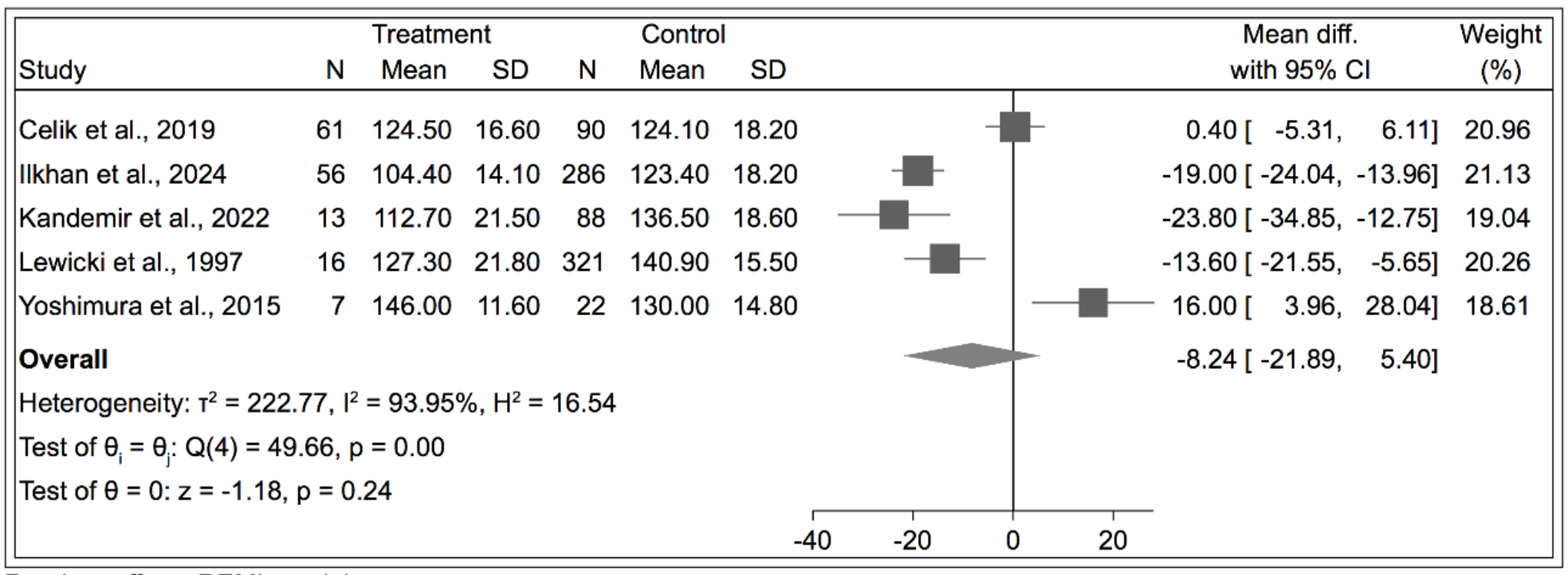


Random-effects REML model

## Supplementary Figure S8. Sensitivity analyses for use of circulatory agents

a) Excluding studies with high risk of bias

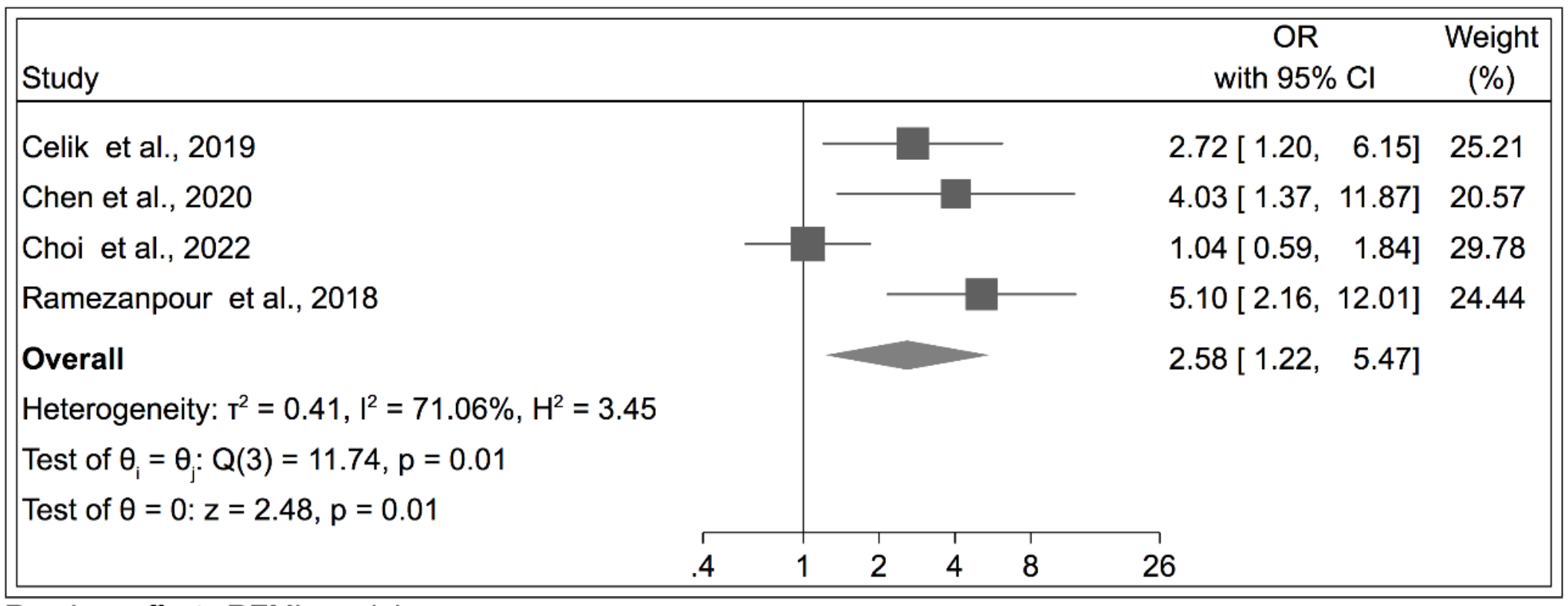


Random-effects REML model

b) Restricting analyses to cross-sectional studies

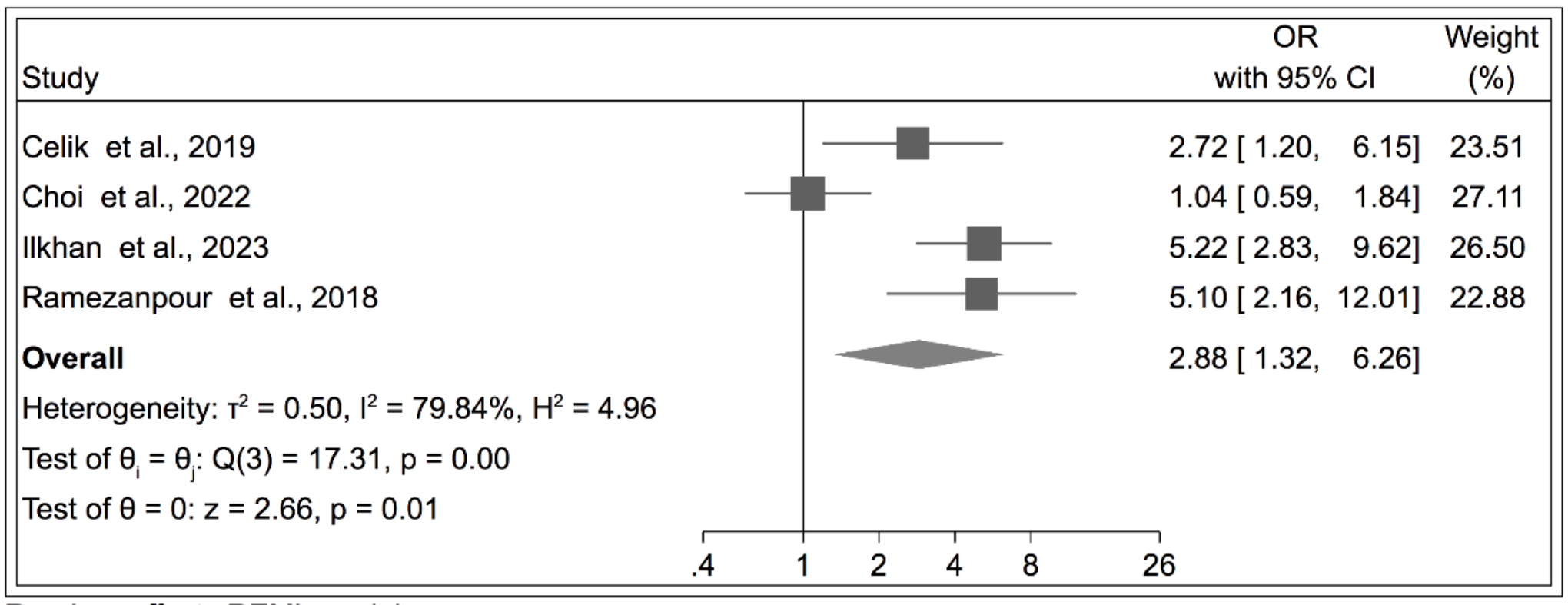


Random-effects REML model

c) Restricting analyses to crude effect estimates

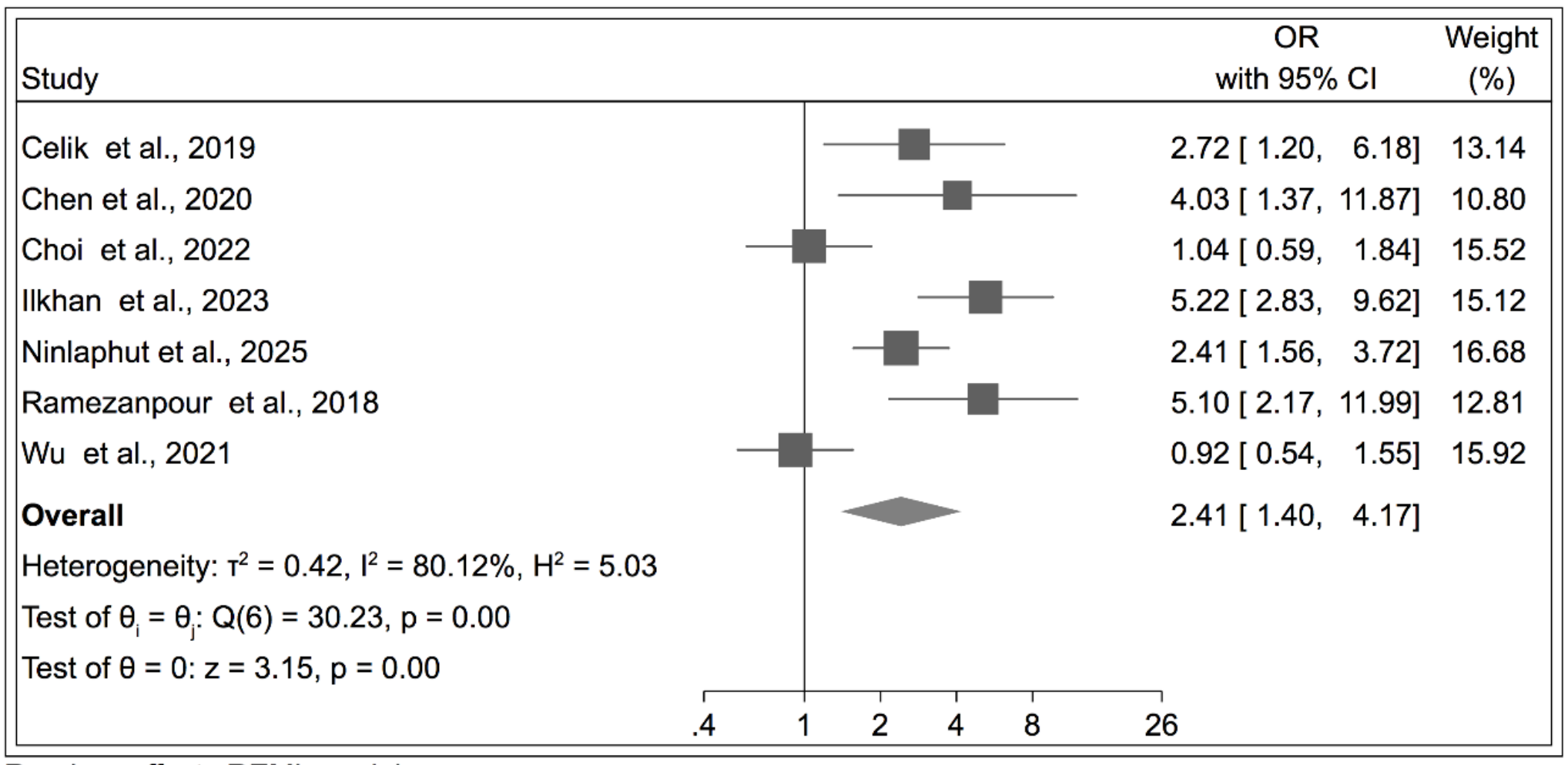


Random-effects REML model

## Supplementary Figure S9. Sensitivity analyses for surgery duration

a) Excluding studies with high risk of bias

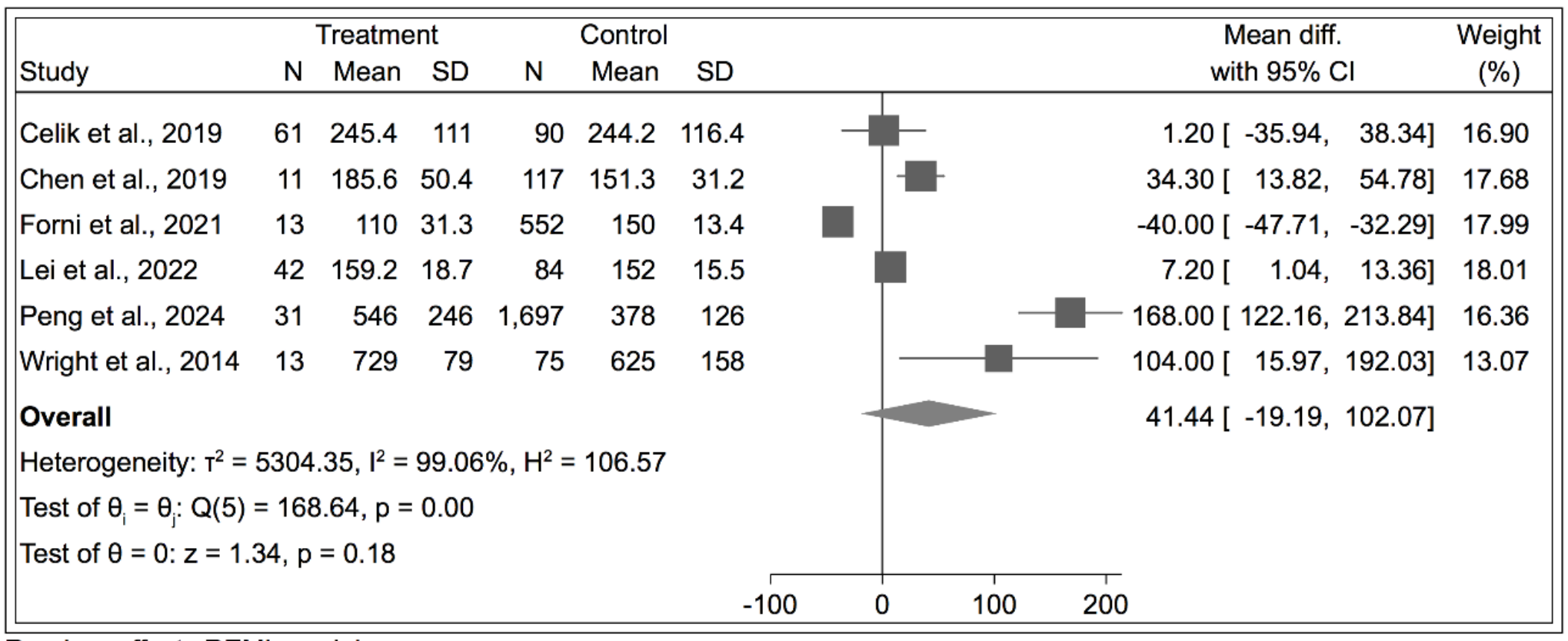


Random-effects REML model

b) Restricting analyses to cross-sectional studies

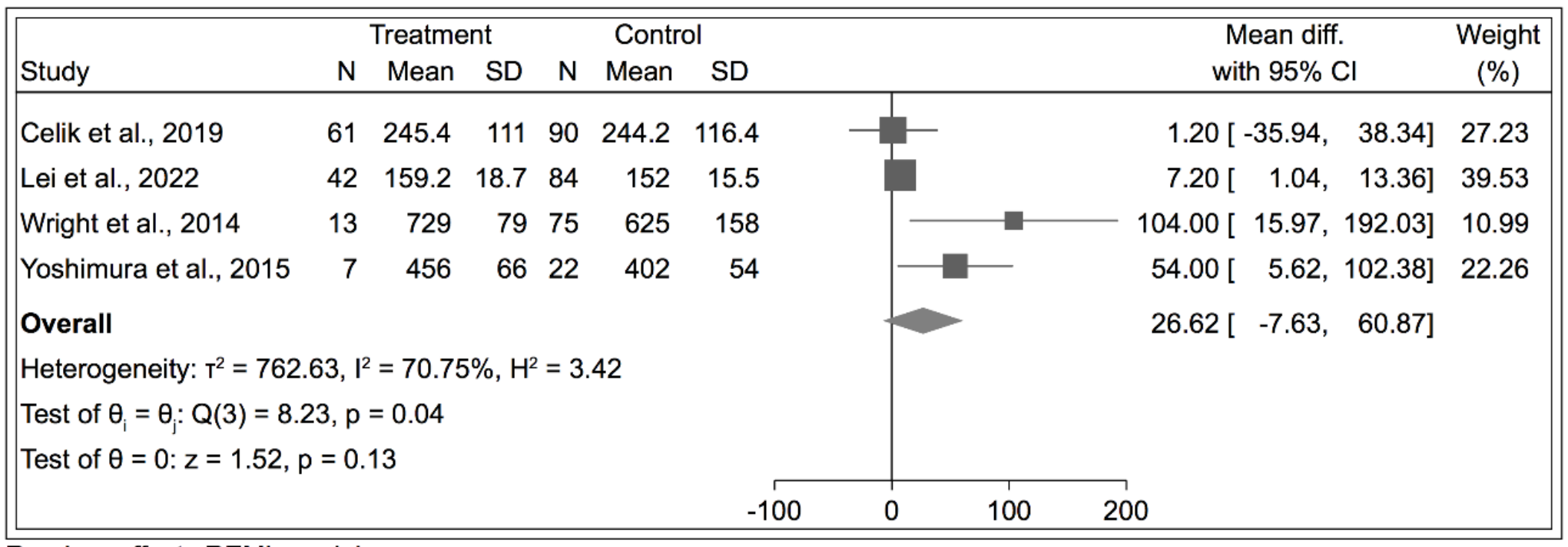


Random-effects REML model
