# Supplementary material for: Risk factors for position-related injuries in adult surgical patients: a systematic review and meta-analysis
Source: Int J Nurs Stud Adv. 2026 Sep 8;11:100676. doi: 10.1016/j.ijnsa.2026.100676 (PMC13598170; doi:10.1016/j.ijnsa.2026.100676)

Supplementary figures S10-S18. Influence analyses

Supplementary Figure S10. Influence analysis for sex

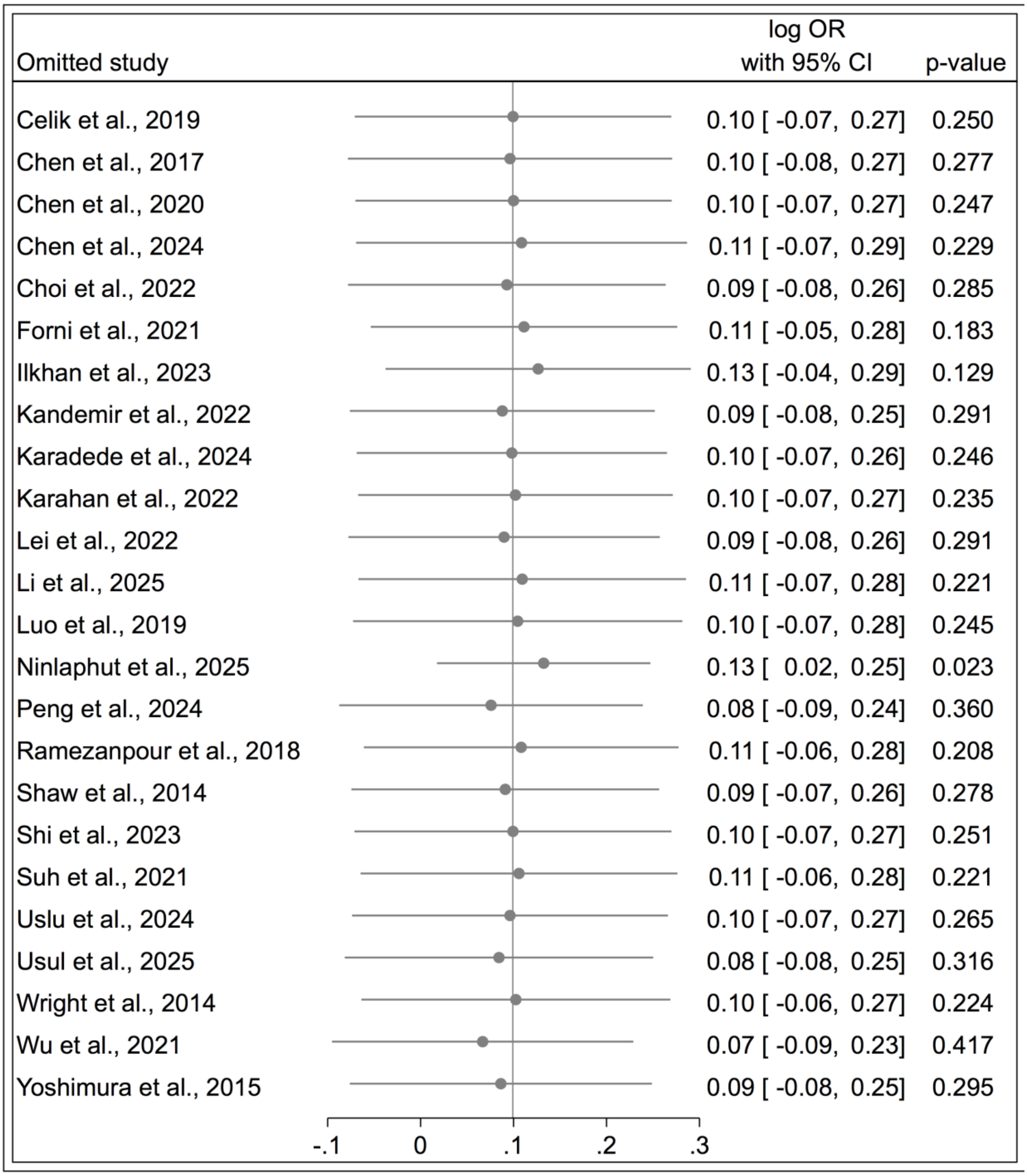

Random-effects REML model

Supplementary Figure S11. Influence analysis for diabetes

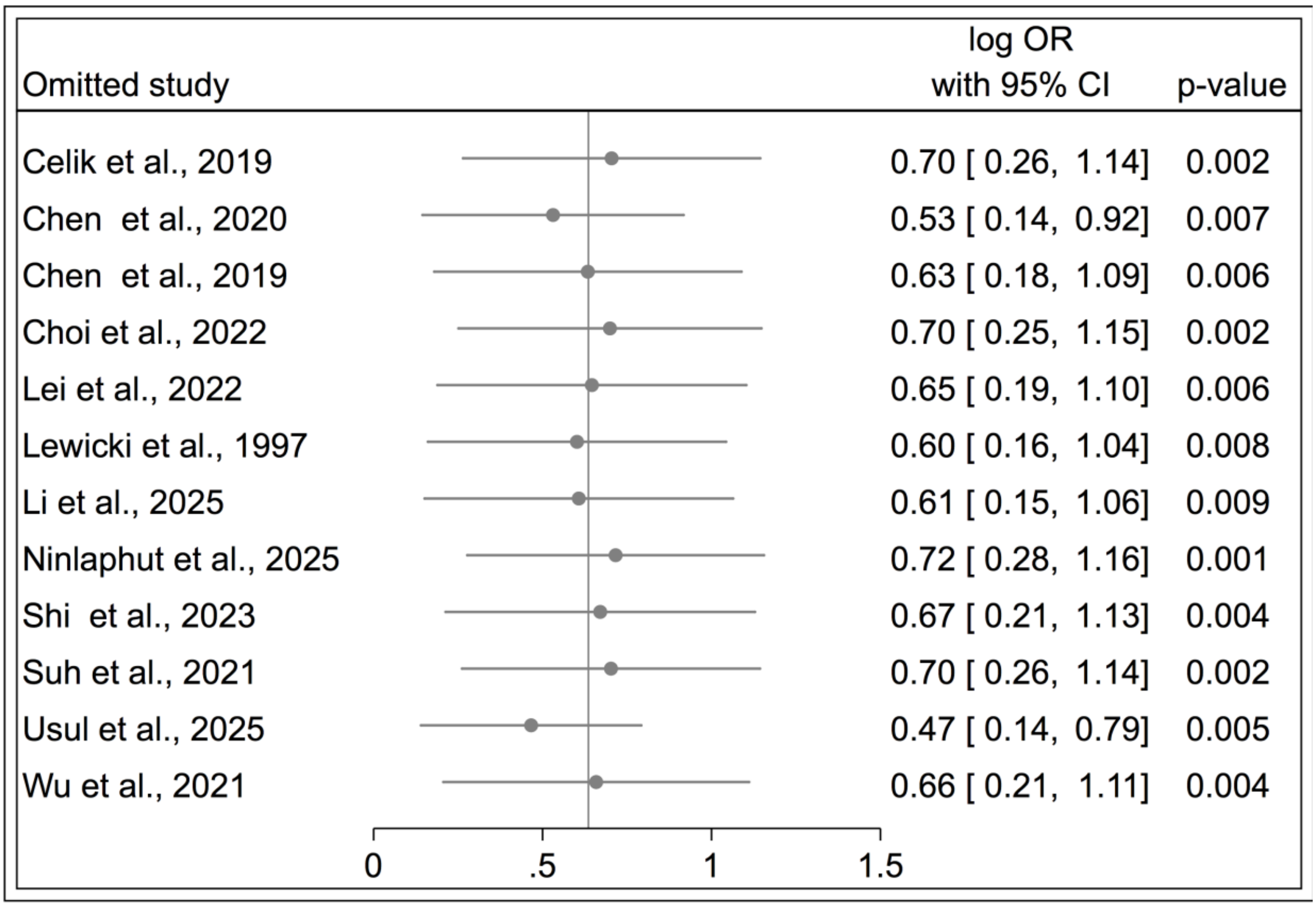

Random-effects REML model

Supplementary Figure S12. Influence analysis for hypertension

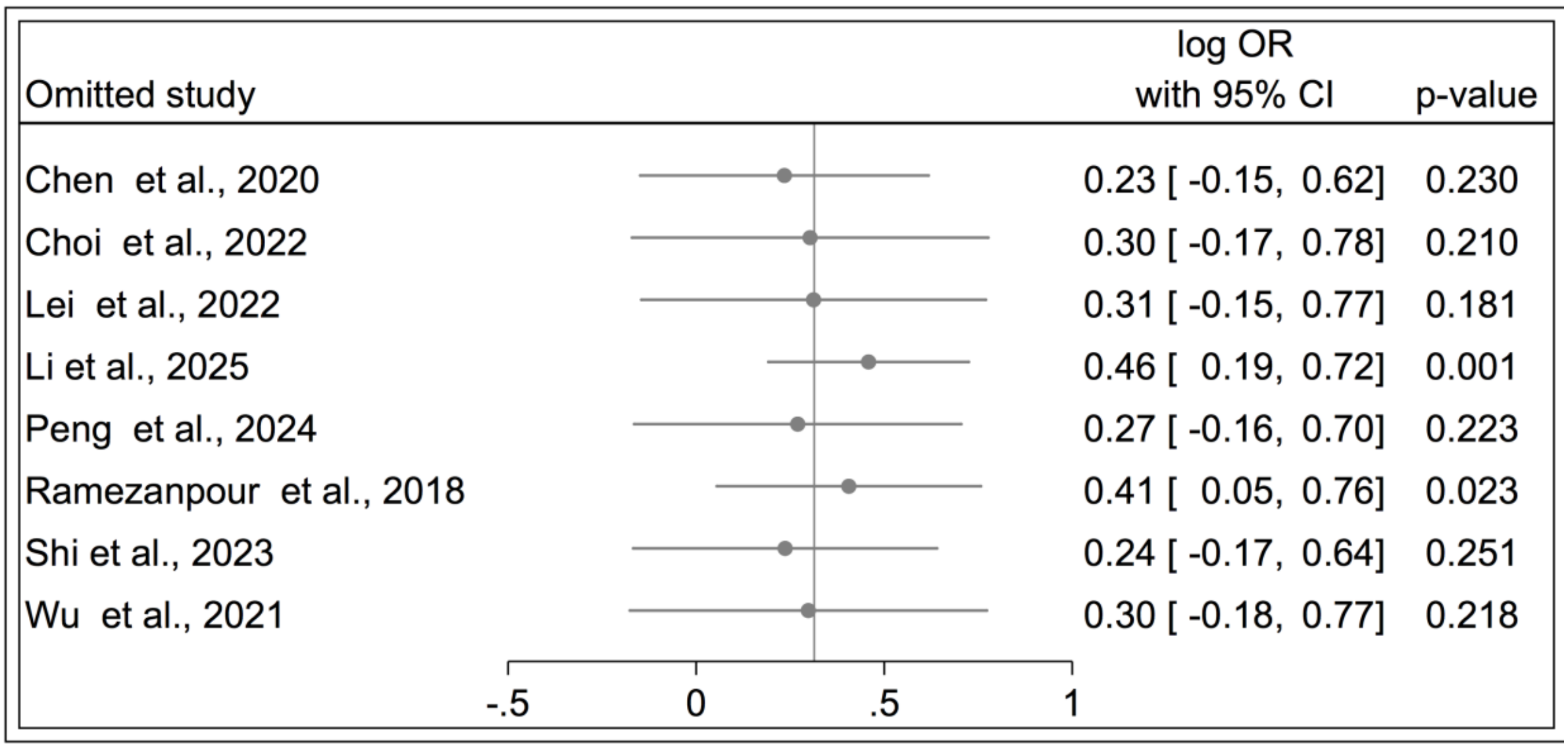

Random-effects REML model

Supplementary Figure S13. Influence analysis for age

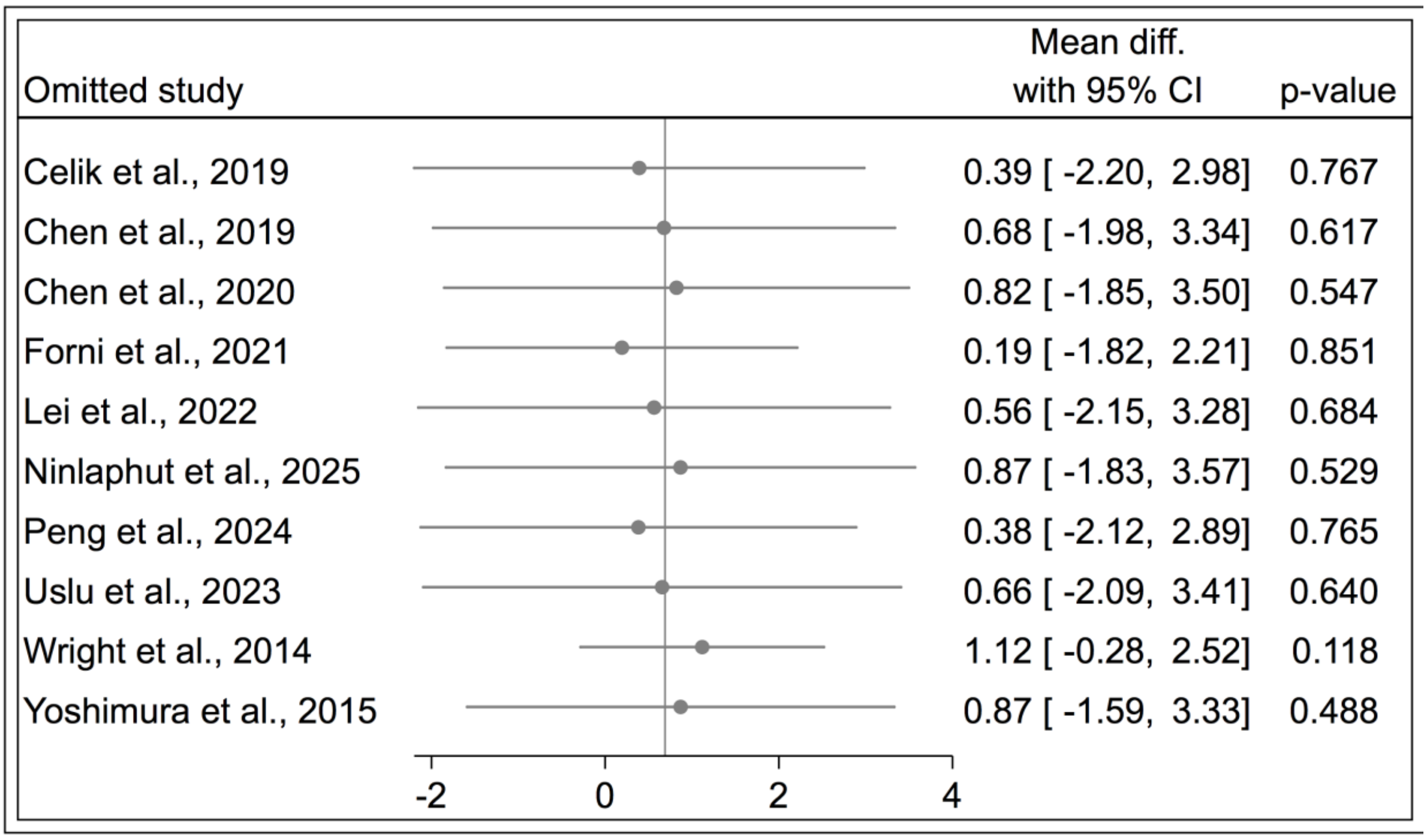

Random-effects Paule–Mandel model

Supplementary Figure S14. Influence analysis for BMI

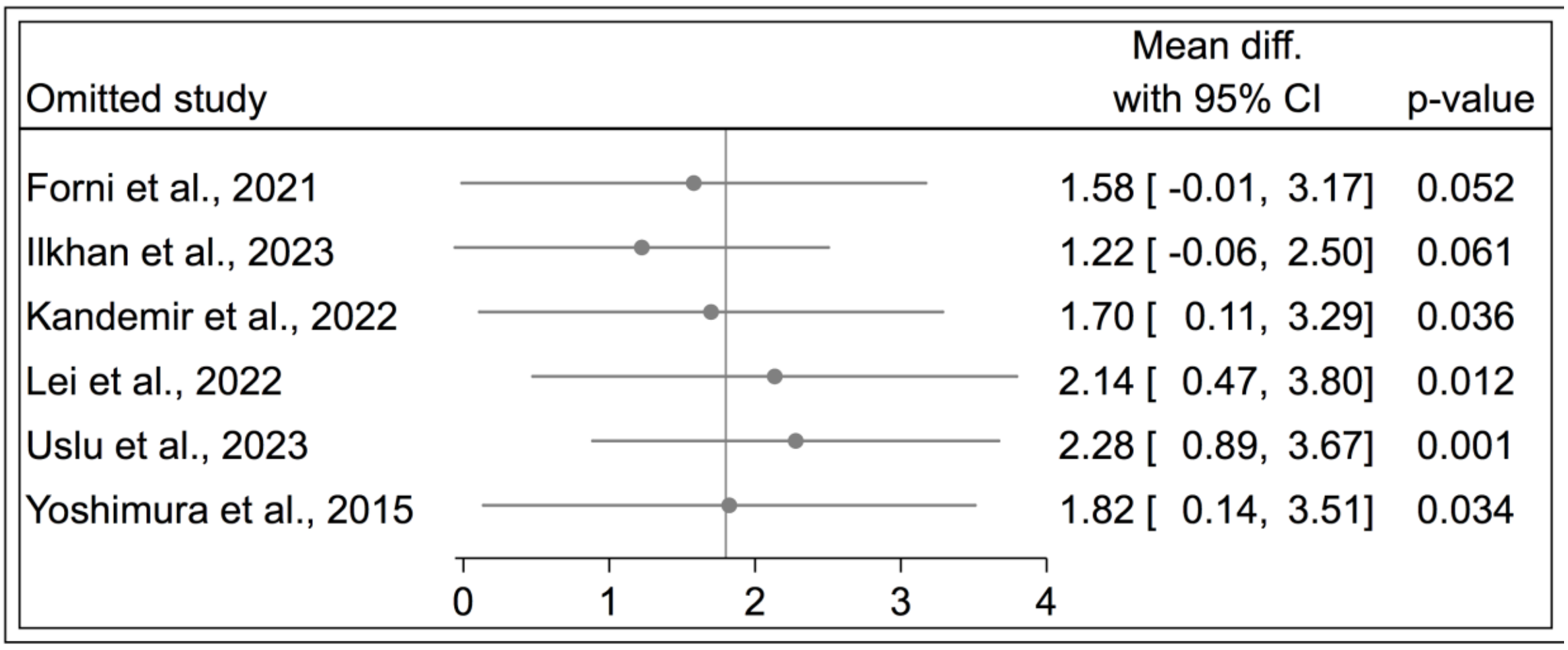

Random-effects REML model

Supplementary Figure S15. Influence analysis for preoperative haemoglobin

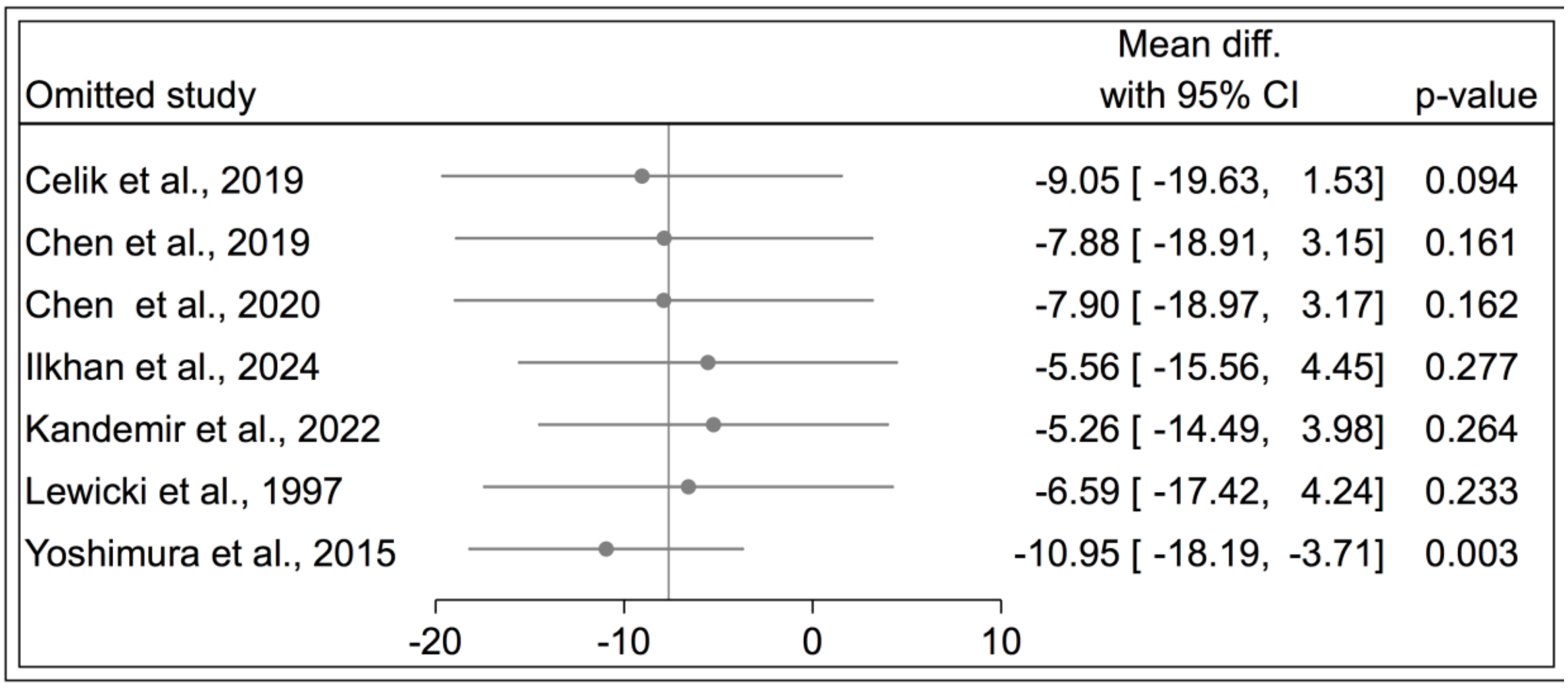

Omitted study	Mean diff. with 95% CI	p-value
Celik et al., 2019	-9.05 [-19.63, 1.53]	0.094
Chen et al., 2019	-7.88 [-18.91, 3.15]	0.161
Chen et al., 2020	-7.90 [-18.97, 3.17]	0.162
Ilkhan et al., 2024	-5.56 [-15.56, 4.45]	0.277
Kandemir et al., 2022	-5.26 [-14.49, 3.98]	0.264
Lewicki et al., 1997	-6.59 [-17.42, 4.24]	0.233
Yoshimura et al., 2015	-10.95 [-18.19, -3.71]	0.003

Random-effects REML model

Supplementary Figure S16. Influence analysis for preoperative albumin

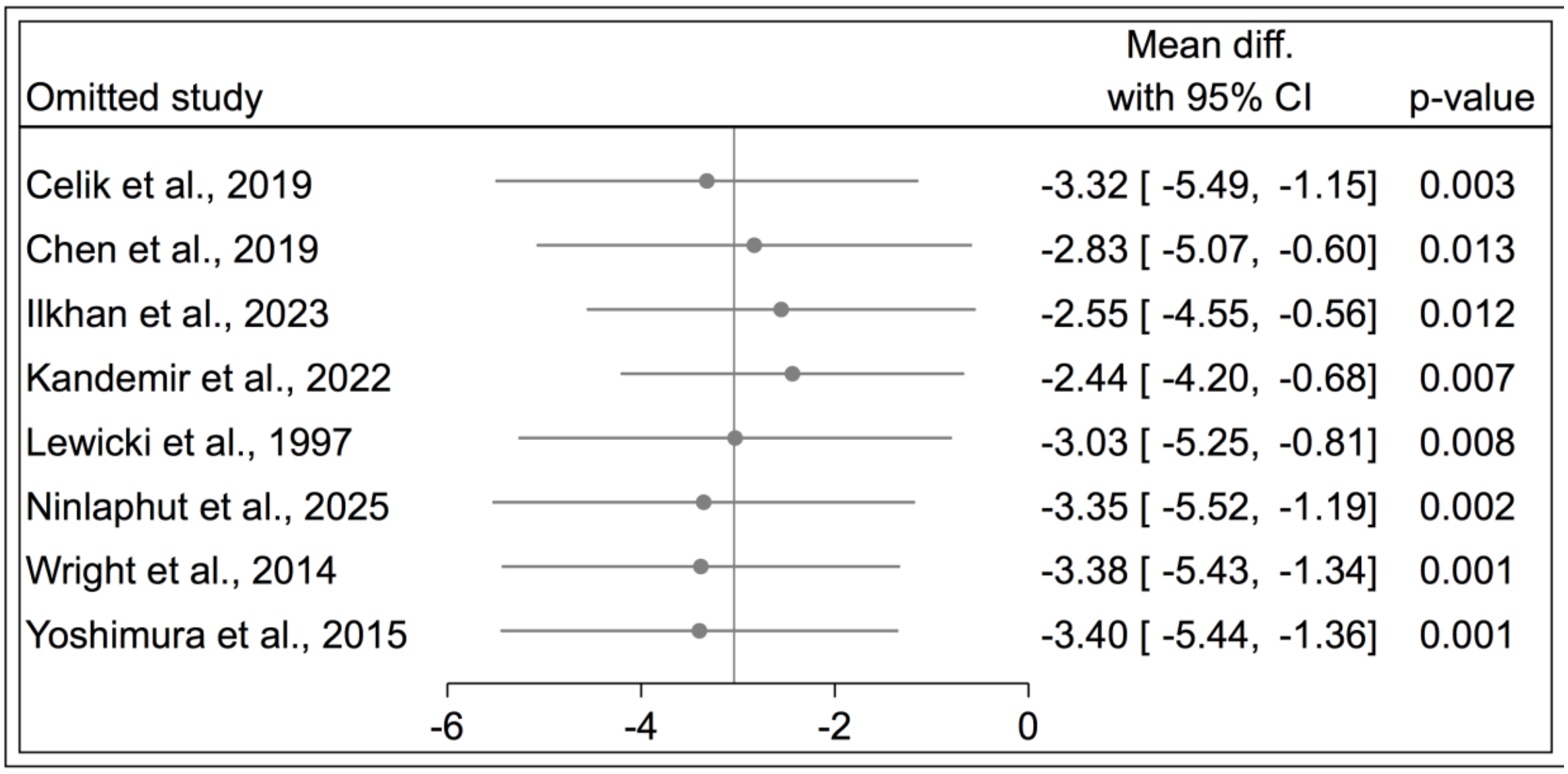

Omitted study	Mean diff. with 95% CI	p-value
Celik et al., 2019	-3.32 [-5.49, -1.15]	0.003
Chen et al., 2019	-2.83 [-5.07, -0.60]	0.013
Ilkhan et al., 2023	-2.55 [-4.55, -0.56]	0.012
Kandemir et al., 2022	-2.44 [-4.20, -0.68]	0.007
Lewicki et al., 1997	-3.03 [-5.25, -0.81]	0.008
Ninlaphut et al., 2025	-3.35 [-5.52, -1.19]	0.002
Wright et al., 2014	-3.38 [-5.43, -1.34]	0.001
Yoshimura et al., 2015	-3.40 [-5.44, -1.36]	0.001

Random-effects REML model

Supplementary Figure S17. Influence analysis for surgery duration

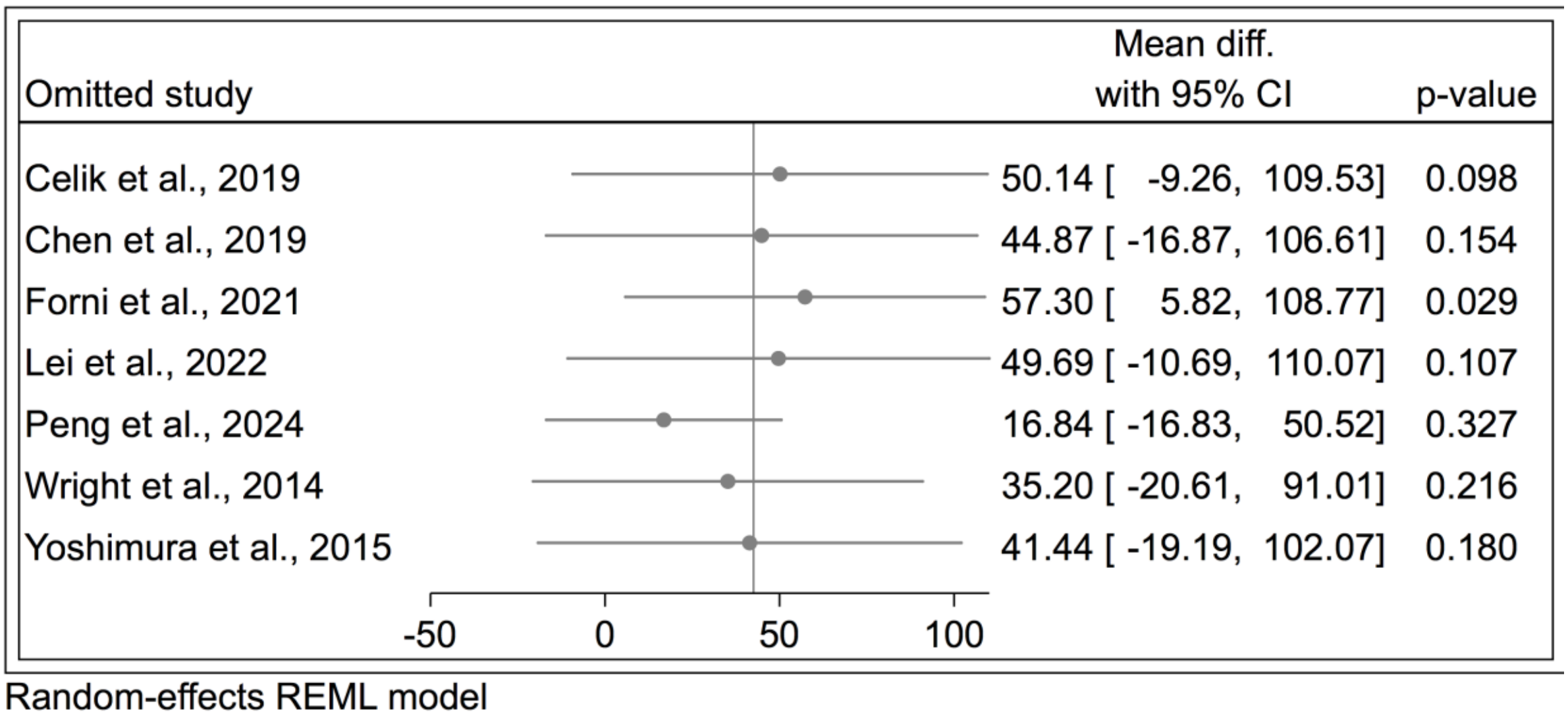

Supplementary Figure S18. Influence analysis for circulatory agents

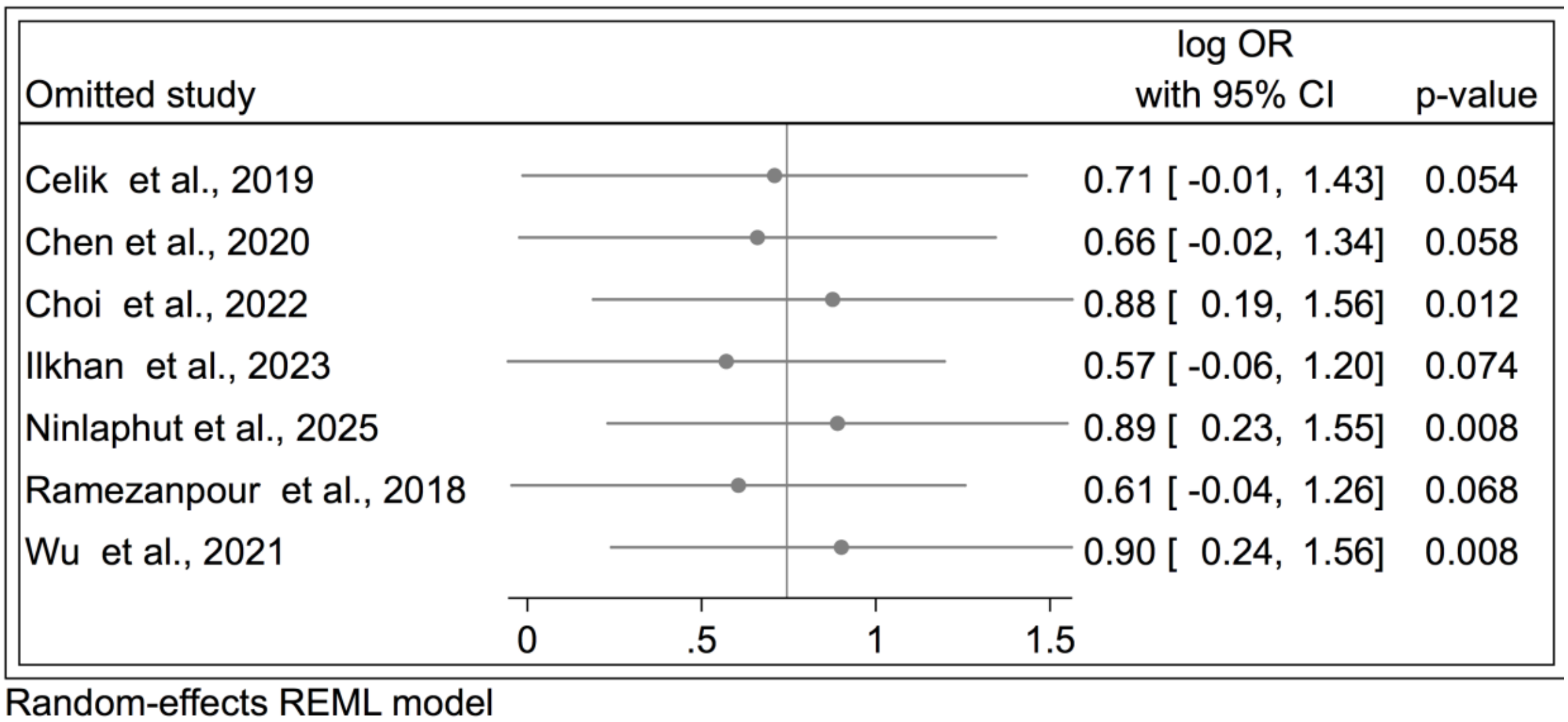

Supplement: Supplementary file 2 [file mmc5.pdf]
