# Supplementary material for: Risk factors for position-related injuries in adult surgical patients: a systematic review and meta-analysis
Source: Int J Nurs Stud Adv. 2026 Sep 8;11:100676. doi: 10.1016/j.ijnsa.2026.100676 (PMC13598170; doi:10.1016/j.ijnsa.2026.100676)

Supplementary figures S19-S21. Funnel plots

Supplementary Figure S19. Funnel plot for sex

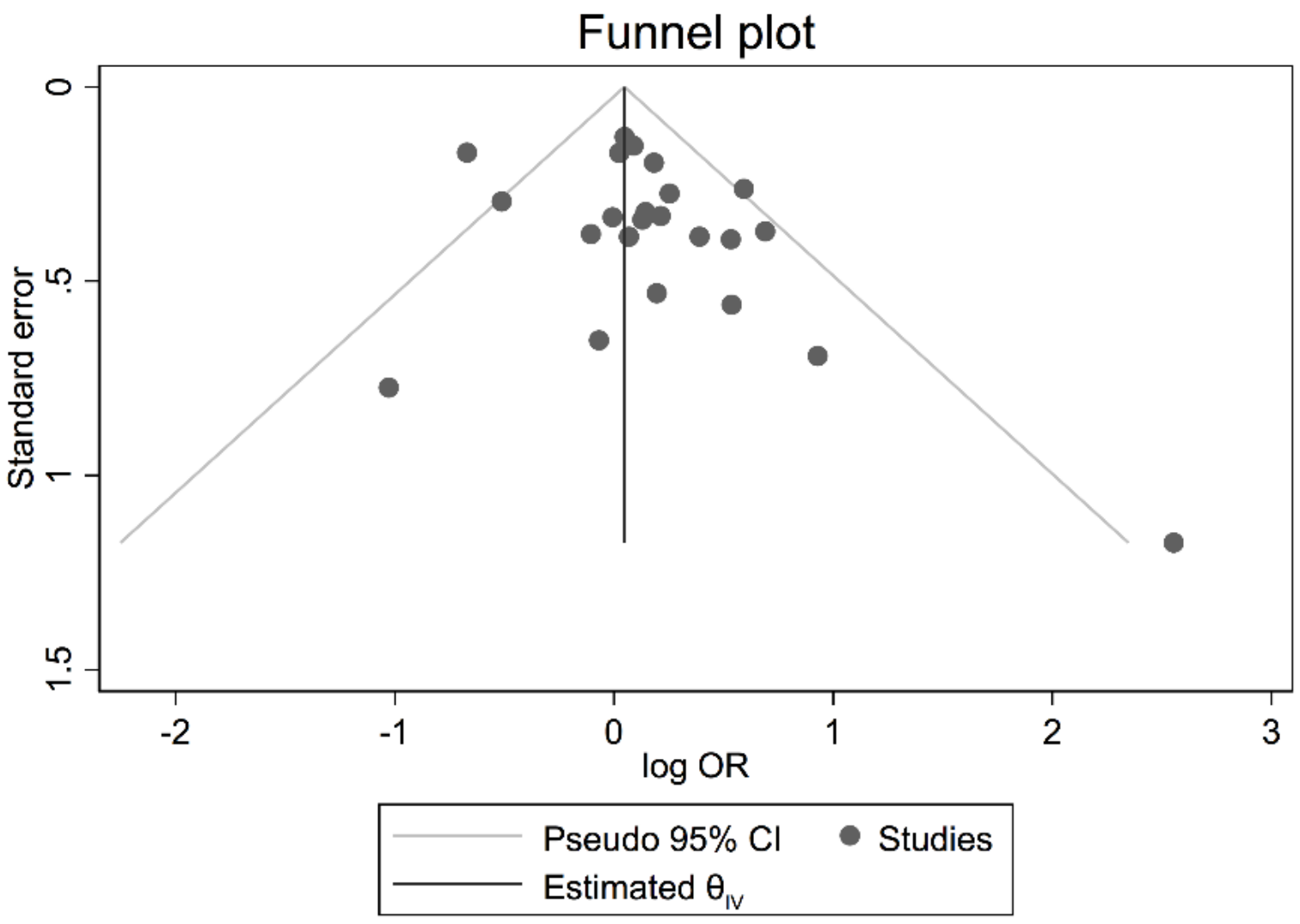

Supplementary Figure S20. Funnel plot for diabetes

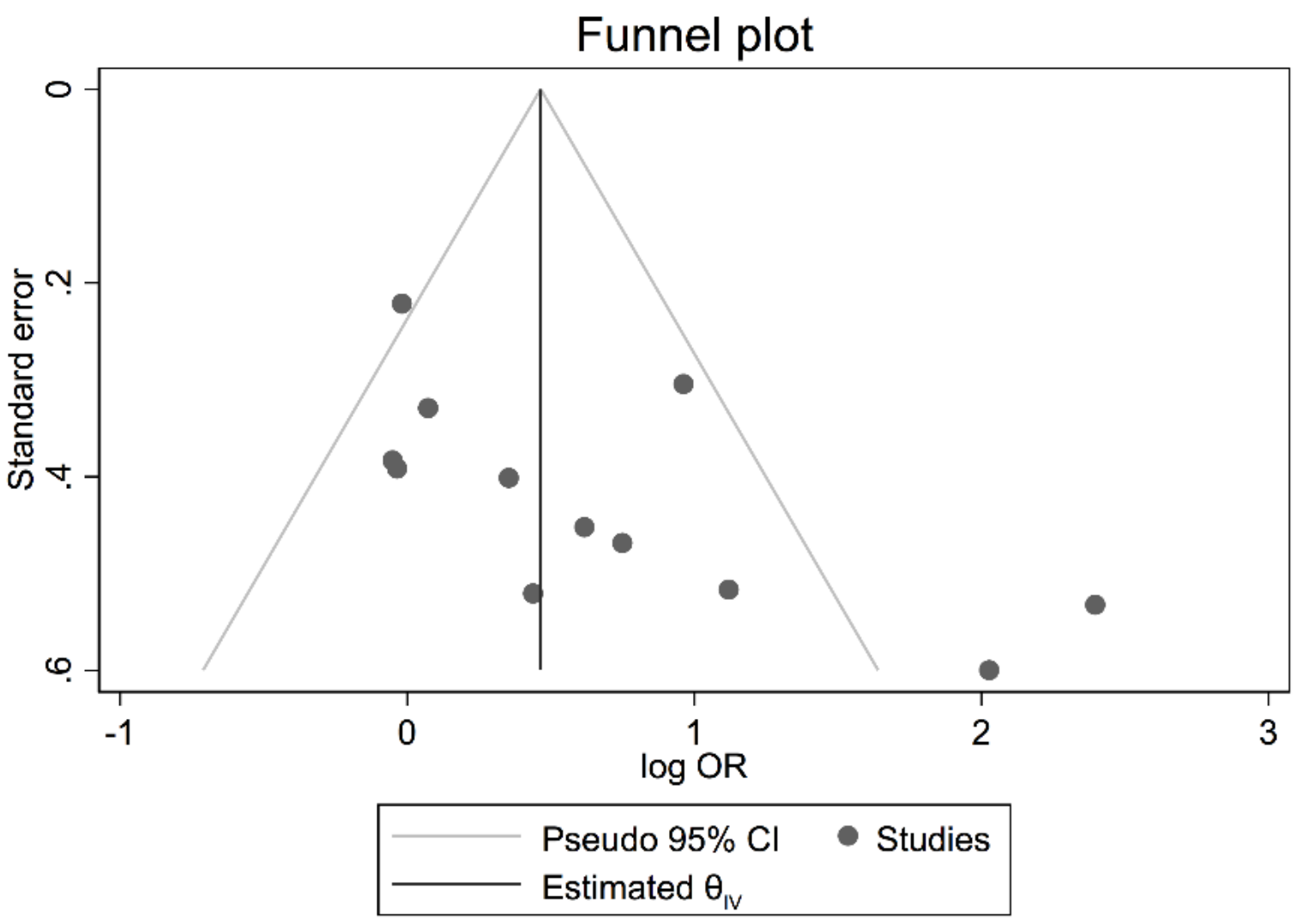

Supplementary Figure S21. Funnel plot for age

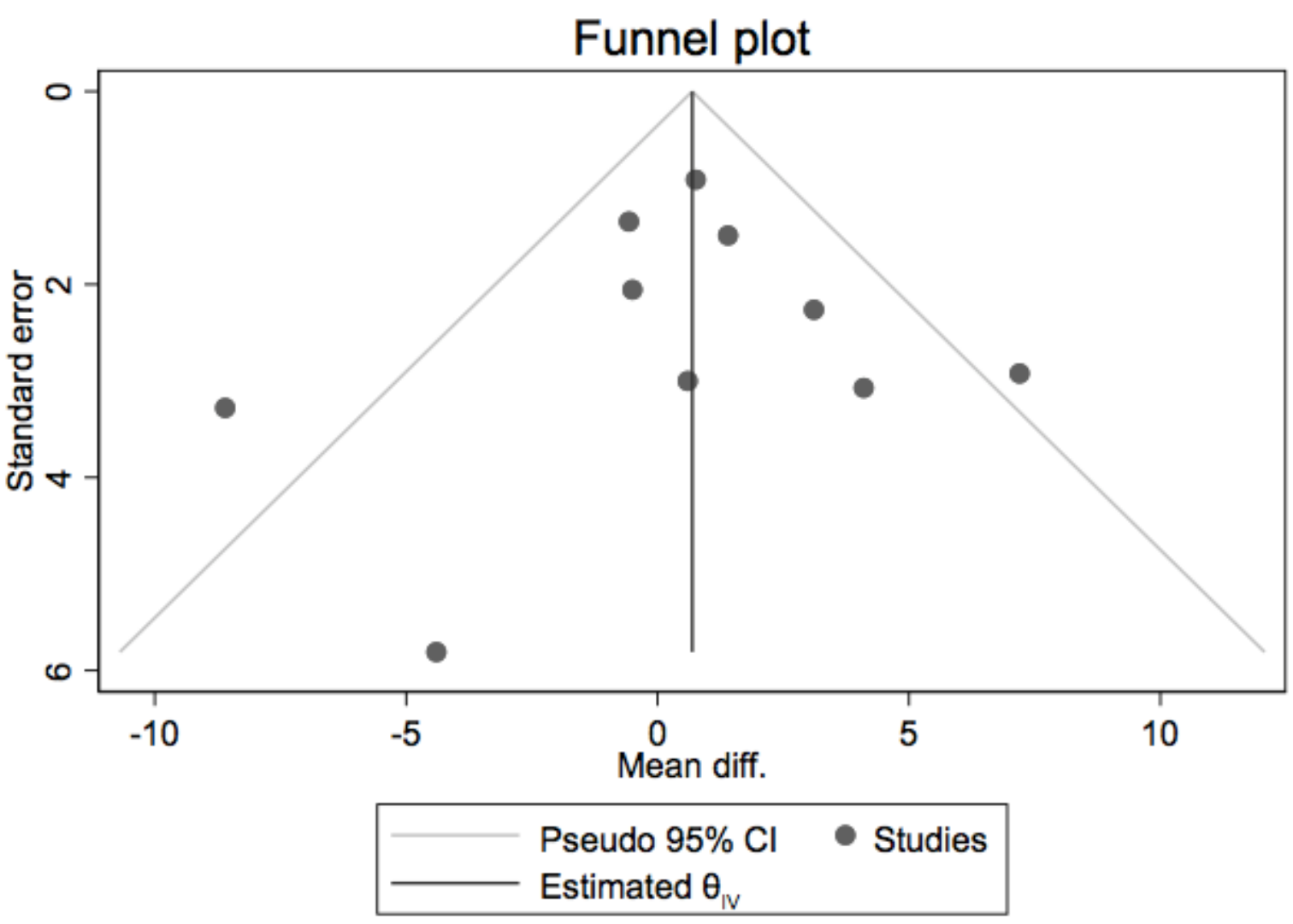

Supplement: Supplementary file 3 [file mmc6.pdf]
