# Supplementary material for: Risk factors for position-related injuries in adult surgical patients: a systematic review and meta-analysis
Source: Int J Nurs Stud Adv. 2026 Sep 8;11:100676. doi: 10.1016/j.ijnsa.2026.100676 (PMC13598170; doi:10.1016/j.ijnsa.2026.100676)
Supplement: Supplementary file 5 [file mmc2.docx]

| **Supplementary Table S1.** Risk of Bias assessment of studies on position-related pressure and peripheral nerve injuries | | | | | | | | | | | | | |
| --- | --- | --- | --- | --- | --- | --- | --- | --- | --- | --- | --- | --- | --- |
| **Reference** | **Selection bias** |  |  | **Performance bias** | **Detection bias** | **Attrition bias** |  | **Reporting bias** | **Confounding** |  | **Precision** |  | **Overall assessment** |
| **Item no.** | **1** | **2** | **3** | **4** | **5** | **6** | **7** | **8** | **9** | **10** | **11** | **12** | **13** |
| **Pressure injuries** | | | | | | | | | | | | | |
| Bulfone et al., 2012 | NA | NA | NA | H | L | L | NA | H | NA | H | H | H | H |
| Buso et al., 2021 | NA | NA | NA | H | L | L | NA | H | NA | U | L | L | H |
| Celik et al., 2019 | NA | NA | NA | H | L | H | NA | L | NA | H | L | H | L |
| Chen et al., 2017 | NA | NA | NA | H | U | L | NA | H | NA | L | L | L | L |
| Chen et al., 2019 | NA | NA | NA | H | H | L | NA | L | NA | L | L | L | L |
| Chen et al., 2020 | NA | NA | NA | L | U | L | NA | L | NA | L | L | L | L |
| Chen et al., 2024 | NA | NA | NA | H | H | L | NA | L | NA | NA | L | H | H |
| Choi et al., 2024 | NA | NA | NA | L | H | L | NA | L | NA | L | L | L | L |
| Connor et al., 2010 | NA | NA | NA | H | L | H | NA | H | NA | L | L | L | H |
| Dong et al., 2021 | L | U | L | H | U | H | NA | H | L | H | H | H | H |
| Forni et al., 2021 | NA | NA | NA | L | L | H | NA | L | NA | L | L | L | L |
| İlkhan & Sucu Dag, 2023 | NA | NA | NA | H | L | NA | NA | H | NA | H | L | H | H |
| Kandemir et al., 2022 | L | L | NA | L | L | H | NA | H | NA | L | L | L | L |
| Karadede et al., 2025 | NA | NA | NA | H | L | NA | NA | H | NA | L | L | L | H |
| Karahan et al., 2022 | NA | NA | NA | H | H | L | NA | L | NA | U | L | H | H |
| Kemp et al., 1990 | NA | NA | NA | H | L | L | NA | H | NA | H | H | U | H |
| Lei et al., 2022 | NA | NA | NA | L | U | H | NA | H | NA | L | L | L | L |
| Lewicki et al., 1997 | NA | NA | NA | L | L | L | NA | L | NA | H | L | H | L |
| Li et al., 2025 | NA | NA | NA | U | U | H | NA | H | NA | L | L | L | H |
| Luo et al., 2019 | NA | NA | NA | H | H | H | NA | L | NA | L | L | L | L |
| Ninlaphut et al, 2025 | NA | NA | NA | H | U | NA | NA | H | NA | L | L | L | H |
| Peng et al., 2024 | NA | NA | NA | L | H | H | NA | L | NA | L | L | L | L |
| Ramezanpour et al., 2018 | NA | NA | NA | L | L | L | NA | H | NA | L | L | L | L |
| Scarlatti et al., 2011 | NA | NA | NA | H | H | L | NA | L | NA | L | L | L | L |
| Shaw et al., 2014 | NA | NA | NA | H | H | L | NA | H | NA | L | L | L | L |
| Shi et al., 2023 | NA | NA | NA | L | H | NA | NA | L | NA | L | L | L | L |
| Suh et al., 2021 | NA | NA | NA | H | U | H | NA | L | NA | L | L | L | L |
| Uslu & Adıgüzel Akbaba, 2024 | NA | L | NA | L | L | H | NA | H | NA | L | L | U | L |
| Usul & Dizer, 2025 | NA | NA | NA | H | H | NA | NA | H | NA | H | L | U | H |
| Wright et al., 2014 | NA | NA | NA | U | U | H | NA | H | NA | L | H | L | L |
| Wu et al., 2021 | NA | NA | NA | U | U | H | NA | H | NA | L | L | L | H |
| Yılmaz & Başlı, 2021 | NA | NA | NA | H | H | L | NA | H | NA | H | L | H | H |
| Yoshimura et al., 2015 | NA | NA | NA | L | L | L | NA | L | H | H | H | H | H |
| **Peripheral nerve injuries** | | | | | | | | | | | | | |
| Behera et al., 2025 | NA | NA | NA | H | H | NA | U | L | NA | H | H | H | H |
| Holtzman et al., 2017 | NA | NA | NA | H | L | L | NA | H | NA | H | H | H | H |
| Koç et al., 2012 | NA | NA | NA | H | H | H | NA | H | NA | H | L | H | H |
| Navarro-Vicente et al., 2012 | NA | NA | NA | H | H | H | NA | L | NA | H | L | H | H |
| Posta et al., 1997 | NA | NA | NA | H | H | H | NA | H | NA | H | H | L | H |
| Zarandona Del Campo et al., 2023 | NA | NA | NA | L | L | NA | NA | H | NA | L | H | L | L |
| Warner et al., 1994a | U | L | U | H | L | H | NA | L | L | L | L | L | H |
| Warner et al., 1994b | U | U | L | H | L | H | NA | H | L | L | L | L | H |
| H = high risk of bias, L = low risk of bias, U = unclear risk of bias, NA = not applicable  Selection bias = items 1-3, Attrition bias = items 6-7, Confounding = items 9-10, Precision = items 11-12 | | | | | | | | | | | | | |
