# Supplementary material for: Risk factors for position-related injuries in adult surgical patients: a systematic review and meta-analysis
Source: Int J Nurs Stud Adv. 2026 Sep 8;11:100676. doi: 10.1016/j.ijnsa.2026.100676 (PMC13598170; doi:10.1016/j.ijnsa.2026.100676)
Supplement: Supplementary file 6 [file mmc3.docx]

| Supplementary Table S2. Meta-analyses of risk factors for pressure injuries | | | | | |
| --- | --- | --- | --- | --- | --- |
| Risk factors for pressure injuries | **No. of studies** | **Pooled estimate** | **Heterogeneity** | | |
| Dichotomous variables |  | **Odds ratio**  **(95% CI)** | **P-value** | **I² (%) ª** | **τ^2 ᵇ^** |
| Sex ᶜ (male/female) | 24 | 1.10  (0.94, 1.30) | 0.23 | 44.11 | 0.06 |
| Diabetes (yes/no) | 12 | 1.89  (1.25, 2.86) | <0.001 | 70.48 | 0.36 |
| Hypertension (yes/no) | 8 | 1.37  (0.93, 2.01) | 0.11 | 61.84 | 0.18 |
| Intraoperative use of circulatory agents (yes/no) | 7 | 2.11  (1.13, 3.92) | 0.02 | 81.45 | 0.56 |
| Continuous variables |  | **Mean difference (95% CI)** | **P-value** | **I² (%)** | **τ^2^** |
| Age (years) | 10 | 0.69  (-1.65, 3.03) | 0.56 | 69.35 | 8.45 |
| BMI (kg/m²) | 6 | 1.80  (0.38, 3.22) | 0.01 | 78.95 | 2.27 |
| Preoperative Haemoglobin (g/L) | 7 | -7.64  (-16.90, 1.62) | 0.11 | 90.27 | 137.36 |
| Preoperative s-albumin (g/L) | 8 | -3.04  (-4.99, -1.09) | <0.001 | 85.45 | 6.40 |
| Surgery duration (min) | 7 | 42.54  (-9.03, 94.11) | 0.11 | 98.68 | 4394.20 |
| Statistical methods are random-effect models for all risk factors; the Paule-Mandel estimator was applied for age, and restricted maximum likelihood (REML) was used for all other variables.  ª The I^2^ parameter represents the percentage of total variation across studies that is attributable to heterogeneity rather than chance.  ᵇ The τ^2^ parameter represents the between-study variance  ᶜ Male sex was predetermined as risk factor | | | | | |
